# Human Staufen1 Associates to MiRNAs Involved in Neuronal Cell Differentiation and is Required for Correct Dendritic Formation

**DOI:** 10.1371/journal.pone.0113704

**Published:** 2014-11-25

**Authors:** Joan Peredo, Patricia Villacé, Juan Ortín, Susana de Lucas

**Affiliations:** 1 Departamento de Biología Molecular y Celular, Centro Nacional de Biotecnología (CSIC), Madrid, Spain; 2 Ciber de Enfermedades Respiratorias (ISCIII), Madrid, Spain; Korea University, Korea, Republic of

## Abstract

Double-stranded RNA-binding proteins are key elements in the intracellular localization of mRNA and its local translation. Staufen is a double-stranded RNA binding protein involved in the localised translation of specific mRNAs during *Drosophila* early development and neuronal cell fate. The human homologue Staufen1 forms RNA-containing complexes that include proteins involved in translation and motor proteins to allow their movement within the cell, but the mechanism underlying translation repression in these complexes is poorly understood. Here we show that human Staufen1-containing complexes contain essential elements of the gene silencing apparatus, like Ago1-3 proteins, and we describe a set of miRNAs specifically associated to complexes containing human Staufen1. Among these, miR-124 stands out as particularly relevant because it appears enriched in human Staufen1 complexes and is over-expressed upon differentiation of human neuroblastoma cells in vitro. In agreement with these findings, we show that expression of human Staufen1 is essential for proper dendritic arborisation during neuroblastoma cell differentiation, yet it is not necessary for maintenance of the differentiated state, and suggest potential human Staufen1 mRNA targets involved in this process.

## Introduction

Post-transcriptional regulatory mechanisms have emerged as an important component of neuronal differentiation [1]. Thus, mRNA localization and its translational repression are essential for cell polarization and the generation of different cell compartments, such as the axon, the dendritic spines, and for dendritic arborisation [2], [3]. Indeed, mRNA binding proteins, which are key players in the transport and local translation of selective transcripts, have emerged as important factors in these processes. This is the case of Staufen, a crucial factor for the localization of specific mRNAs, such as *oskar* and *bicoid* in the fly early development [4] or *prospero* in the neuronal cell fate [5], as well as the Fragile X Mental retardardation protein (FMRP), mutation of which causes a common form of mental disability and autism [6]–[8].

Staufen is a double-stranded RNA binding protein first identified in *D. melanogaster*. In mammals two homologous proteins Staufen1 (Stau1) and Staufen2 (Stau2) have been characterized. Four different alternative-spliced isoforms have been identified for Stau1 that correspond to two protein sizes, 55 kDa and 63 kDa. Human Stau1 (hStau1) localizes in the endoplasmic reticulum and polysomes and forms large ribonucleoprotein complexes called RNA granules [9]–[11]. These granules were originally identified as motile macromolecular structures in neurons, which move along microtubules within dendrites [3]. Interestingly, Stau1 complexes have a dendritic localization in hippocampal rat cells and colocalize with cytoskeleton and transport related proteins, such as kinesin and dynein, suggesting a role for mammalian Stau1 in the transport and localized translation of mRNAs in this cell type [9], [12], [13]. On the other hand, the *D. melanogaster* Staufen RNA granules have been shown to associate to typical P-body proteins of the RNA-induced silencing complex (RISC), such as DCP1, Ago2 or Me31B (called RCK/p54 in humans) [14]. The RISC regulates the translation and degradation of mRNAs mediated by miRNAs. Proteins from the Argonaut family, such as Ago1 to Ago4 form the nucleus of the complex but only Ago2 binds directly miRNAs and bears the endonucleolitic activity [15], [16]. miRNAs are small RNAs 19 to 22 nt in length, that derive from the much longer capped and polyadenylated primary miRNAs (pri-miRNAs) [17]. The nuclear RNA endonuclease Drosha processes these transcripts to generate a second precursor 65 to 70 nt in size (pre-miRNAs) [18], that is transported to the cytoplasm and further processed by Dicer to produce the mature miRNA. The miRNAs are partially complementary to mRNA targets and regulate their stability and translation [19], [20]. In this way, miRNAs control multiple cell processes such as inflammation [21], cell proliferation and cancer [22], [23] or neuronal differentiation [24].

The observation that Staufen RNA granules in *D. melanogaster* contain elements of the RISC [14] suggests that the mRNAs included in them could be repressed by miRNA-mediated mechanisms. In this report, we analyzed the interplay of hStau1 and the miRNA-mediated repression of translation. We show the association of hStau1 with the Ago components of the RISC and identify miR-124 and miR-9 as the miRNAs preferentially associated to hStau1 RNA granules. In agreement with these findings we report the essential role of hStau1 during *in vitro* differentiation of human neuroblastoma cells.

## Materials and Methods

### Biological materials

The plasmids pC-TAP and pChStau-TAP were previously described [12], [25]. Ago1-HA-Flag, Ago2-HA-Flag and Ago3-HA-Flag, as well as GFP-HA-Flag [16], were provided by Addgene. The HEK293T cell line [26] was provided by A. Portela. The SH-SY5Y cell line was obtained from the ECACC (cat. N° 94030304). Polyclonal rabbit antisera specific for hStaufen1 or influenza virus NP were previously described [10], [27]. Monoclonal antibodies against Ago2, RCK/p54 and HA were purchased from Abcam, MBL and Covance, respectively.

### Cell culture and transfection

Culture of HEK293T and SH-SY5Y cells was performed as described [28], [29]. Briefly, SH-SY5Y cells were seeded on dishes previously incubated with matrigel (BD bioscience) for 1 hour and grown in RPMI (GIBCO) containing 10% bovine foetal serum. Neuroblast differentiation was performed incubating the cells with DMEM 1% bovine foetal serum and 10 µM retinoic acid for 5 days. Then, the medium was discarded and the cells were incubated with Neurobasal medium (GIBCO) containing 1% bovine foetal serum, 2 mM dbAMPc (Sigma), 50 ng/ml BDNF (Alomone), B-27 supplement (GIBCO), 20 mM KCl (MERCK) and 2 mM Glutamax (GIBCO).

Transfection of HEK293T cells was carried out with 25 µg of the corresponding plasmid per 150 mm dish, using the calcium-phosphate method [30] as described [12]. After incubation for 24 h at 37°C, the cells were washed with PBS, collected, centrifuged for 5 minutes at 1500 rpm and 4°C and used for RNA or protein extraction.

### Purification of tagged hStau1 protein

For TAP purification, cell extracts were obtained by lysis in a buffer containing 50 mM Tris-HCl, 100 mM NaCl, and 5 mM EDTA, pH 7.5 (TNE), 0.5% NP-40, 1 mM dithiothreitol (DTT), human placental RNase inhibitor (HPRI) (40 U/ml), and the complete protease inhibitor cocktail (Roche) for 30 min at 4°C. The supernatant was centrifuged at 10,000 rpm for 10 min and 4°C. The lysates were incubated with IgG-Sepharose (GE Healthcare) for 12 h at 4°C. The resin was washed 10 times with 10 resin volumes of IPP-150 (150 mM NaCl, 10 mM Tris-HCl, 0.1% NP40, pH 8.0) buffer and five times with 50 mM Tris-HCl, pH 8, 0.5 mM EDTA, 1 mM DTT. The complexes bound to the resin were digested with 1 U of tobacco etch virus (TEV) protease per 10^7^ cells for 3 h at room temperature. The supernatant was collected, mixed with five washes of the resin with IPP150-CBB (150 mM NaCl, 10 mM Tris-HCl, 0.1% NP40, 10 mM 2-mercaptoethanol, 1 mM Mg(AcO)2, 1 mM imidazole, 2 mM CaCl2, pH 8.0) buffer and incubated with calmodulin-agarose resin (Stratagene) for 12 h at 4°C. The resin was washed 10 times in IPP150-CBB buffer and eluted in a buffer containing 10 mM Tris-HCl, pH 8, 0.1% NP-40, 10 mM β-mercaptoethanol, 1 mM imidazole, and 3 mM EGTA. The purified proteins were analysed by polyacrylamide gel electrophoresis and Western blotting.

### RNA analyses

Both hStau1-associated RNA and RNAs from gel filtration fractions were obtained by treatment with 0.2 mg/ml proteinase K-0.5% SDS in TNE buffer for 30 min at 37°C. After phenol extraction, RNAs were precipitated with 2 volumes of ethanol and 20 µg of glycogen (Roche). Total RNA from cell extracts was obtained using Trizol (Invitrogen) following the manufacturer's instructions. RNAs were dephosphorylated with Shrimp Alkaline Phosphatase (USB-Affimetrix) for 30 minutes at 37°C and subsequently phosphorylated by incubation with T4 Polynucleotide Kinase in the presence of 1 µM γ-^32^P-ATP. The integrity, pattern and size of the Stau-associated RNAs were analyzed in 4% and 15% polyacrylamide-urea gels. The integrity of each RNA preparation was tested using the Agilent 2100 Bioanalyzer.

### Microarray analysis

cDNA was synthesized from 4 µg of RNA using an oligodeoxythymidylic acid 24 nt primer with a T7 polymerase promoter site added to the 3′end. Following second-strand cDNA synthesis, the double-stranded cDNA was purified and used as template in the subsequent *in vitro* transcription (IVT) reaction. This *in vitro* transcription was performed using One-cycle target labelling and control reagents (Affymetrix) to produce biotin labelled cRNA. The biotinylated cRNAs were then cleaned up, fragmented (35–200 bases), and hybridized to the Human Genome U133 Plus 2.0 chip, containing more than 54000 transcripts and 38500 well characterized human genes (Affymetrix). Each sample was added to a hybridization solution containing 100 mM 2-(N-morpholino) ethanesulfonic acid, 1 M Na+, and 20 mM of EDTA in the presence of 0.01% of Tween-20 to a final cRNA concentration of 0.05 µg/ml. Hybridization was performed for 16 h by incubating 200 µl of the sample to MOE 430 2.0 chips at 45°C. Each microarray was stained with streptavidin-phycoerythrin in a Fluidics station 450 (Affymetrix) and scanned at 11 µm resolution in a GeneChip Scanner 3000 7G System (Affymetrix). Data analyses were performed using GeneChip Operating Software (GCOS). Three biological replicates for each condition were independently hybridized. Microarray analysis was performed using the affylmaGUI R package [31]. Robust Multi-array Analysis (RMA) algorithm was used for background correction, normalization and expression levels summarization [32]. Next, differential expression analysis was performed with the Bayes t-statistics from the linear models for Microarray data (Limma), included in the affylmGUI package. P-values were corrected for multiple testing using the Benjamini-Hochberg's method (False Discovery Rate) [33]. Genes were considered as expressed differentially when the corrected P values were <0.05 (or <0.01 where specified). In addition, only genes with a fold change higher than two were considered for further analysis. Microarray data have been deposited in GEO (reference GSE61732).

### RT-PCR

The screening of miRNAs was performed using Multiplex RT-qPCR for TaqMan MicroRNA Assays card A v.2 following the manufacturer instructions (Applied Biosystem). To standardize the results from the various replicate assays we used the accumulation of U6 RNA, that is present in total cell RNA but should serve as a negative control in TAP-associated or hStau1-associated RNAs. For individual miRNA quantification, 10 ng of each RNA tested were used for RT with specific TaqMan miRNA loop-primers for miR-124, miR-149, miR-24, miR-339, miR-345, miR-9, miR-93 or miR-147a and Taqman miRNA RT Master Mix (Applied Biosystem). Next, TaqMan qPCR were carried out by Universal Master Mix II no UNG, following the manufacturer instructions.

### Protein analyses

Gel filtration was performed as previously described [12]. Briefly, cell extracts were applied to a Sephacryl S400 resin equilibrated in 50 mM Tris-HCl, 100 mM NaCl, and 5 mM EDTA, pH 7.5 (TNE), 0.5% NP-40, 1 mM DTT, at a sample to bed volume ratio of 1∶100. The column was previously calibrated with catalase, purified influenza virion ribonucleoproteins (5.5–2.4 MDa) and purified recombinant influenza ribonucleoproteins (0.75 MDa) [27]. The localization of hStau1 complexes, influenza ribonucleoproteins and other cell markers was revealed by Western blot as described previously [10]. For immunofluorescence, the cell cultures were fixed for 20 min with 3% paraformaldehyde and permeabilised with 0.5% Triton X100 in PBS for 5 min. The preparations were blocked during 1 h with 2% foetal bovine serum in PBS and incubated for 1 h with the primary antibody diluted in 0.1% foetal bovine serum in PBS. After washing with PBS, the preparations were incubated with Alexa 546-, 488- or 647-labelled secondary antibodies, mounted in Prolong and visualised with a Leica TCS SP5 microscope. Optical sections were acquired and processed with Leica LASAF software. Morphology analysis of neuroblasts was performed using Image J software including the Neuron J pluggin [34].

### Bioinformatic analyses

Validated mRNA targets for the identified miRNAs were searched using Genecodis Web server (http://genecodis.cnb.csic.es/) [35]–[37]. Predicted targets were searched using two different algorithms, Targetscan (http://www.targetscan.org) [38] and DianaLab (http://diana.cslab.ece.ntua.gr/?sec=home) [39], [40].

## Results

### Human Staufen1 protein associate to specific miRNAs

**Figure 1:**
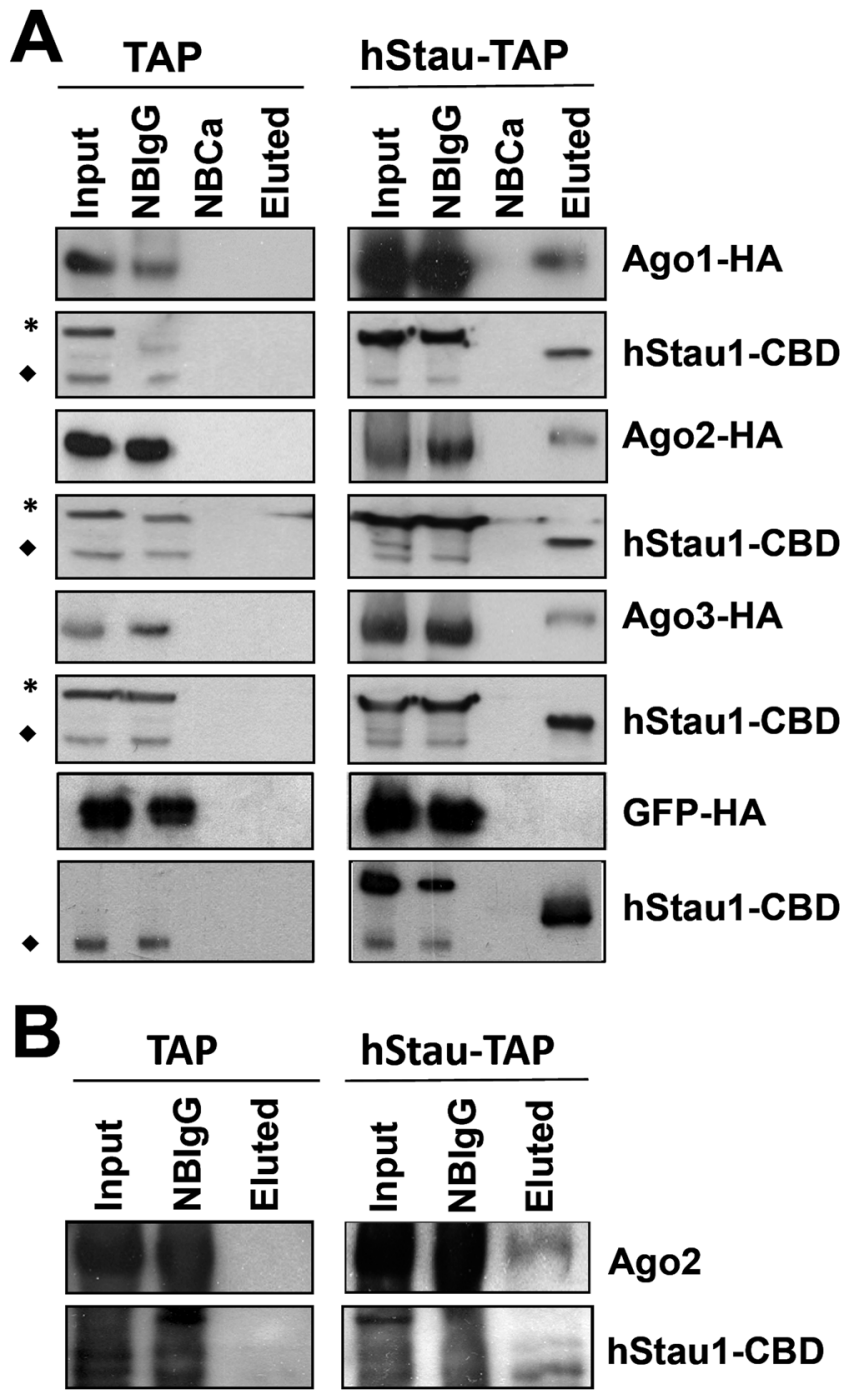
hStaufen1 complexes are associated to the RISC proteins. (A) Cultures of HEK293T cells were transfected with pChStaufen1-TAP (hStau-TAP) or pC-TAP (TAP) and either Ago1-HA, Ago2-HA, Ago3-HA or GFP-HA and soluble extracts were used for TAP purification. The purified complexes were analysed using antibodies specific for HA or hStau1. Total extract (Input), not bound to IgG (NBIgG), not bound to calmodulin (NBCa) and eluted with EGTA (Eluted) are shown. (B) Cultures of HEK293T cells were transfected with pChStaufen1-TAP or pC-TAP and the purified complexes were analysed with antibodies specific for hStau1 or Ago2. Total extract (Input), not bound to IgG (NBIgG) and eluted by digestion with TEV (Eluted) are shown. The mobilities of the Ago or GFP proteins or hStaufen-CBD are indicated to the right. Stars mark unspecific cross-reaction bands detected with the anti-hStau1 antibody and diamonds indicate endogenous hStau1 protein.

As the Staufen RNA granules in *D. melanogaster* colocalise with elements of the RISC [14] we tested whether hStau1 also associates with the miRNA-dependent RNA silencing machinery. To that aim we co-expressed in HEK293T cells a TAP-tagged version of hStau1 (55 kDa isoform) with each of the Ago1 to Ago3 proteins, or GFP as a negative control, containing a C-terminal HA tag. Although these cells were not derived from neural tissue [41], they show some phenotypic characteristics in common with neurons [42], [43] and are very amenable for transfection studies. After purification of hStau1 complexes by the two-steps TAP affinity chromatography, the presence of the Ago proteins or GFP was analysed by Western blot with anti-HA antibodies. The results are presented in Fig. 1A and show that each of the Ago proteins tested associated to purified hStau1 complexes whereas GFP did not, and they were not detectable in control purifications in which the TAP tag was expressed as a negative control. To verify that endogenous Ago proteins also associate to hStau1 complexes we chose Ago2, since it directly binds miRNAs [16]. As presented in Fig. 1B, Ago2 is associated to hStau1 complexes but not to control TAP purifications. With these analyses we cannot distinguish whether Ago protein association to hStau1 complexes is mediated by protein-protein interactions or depend on the association of these members of the RISC complex to specific RNAs.

**Figure 2:**
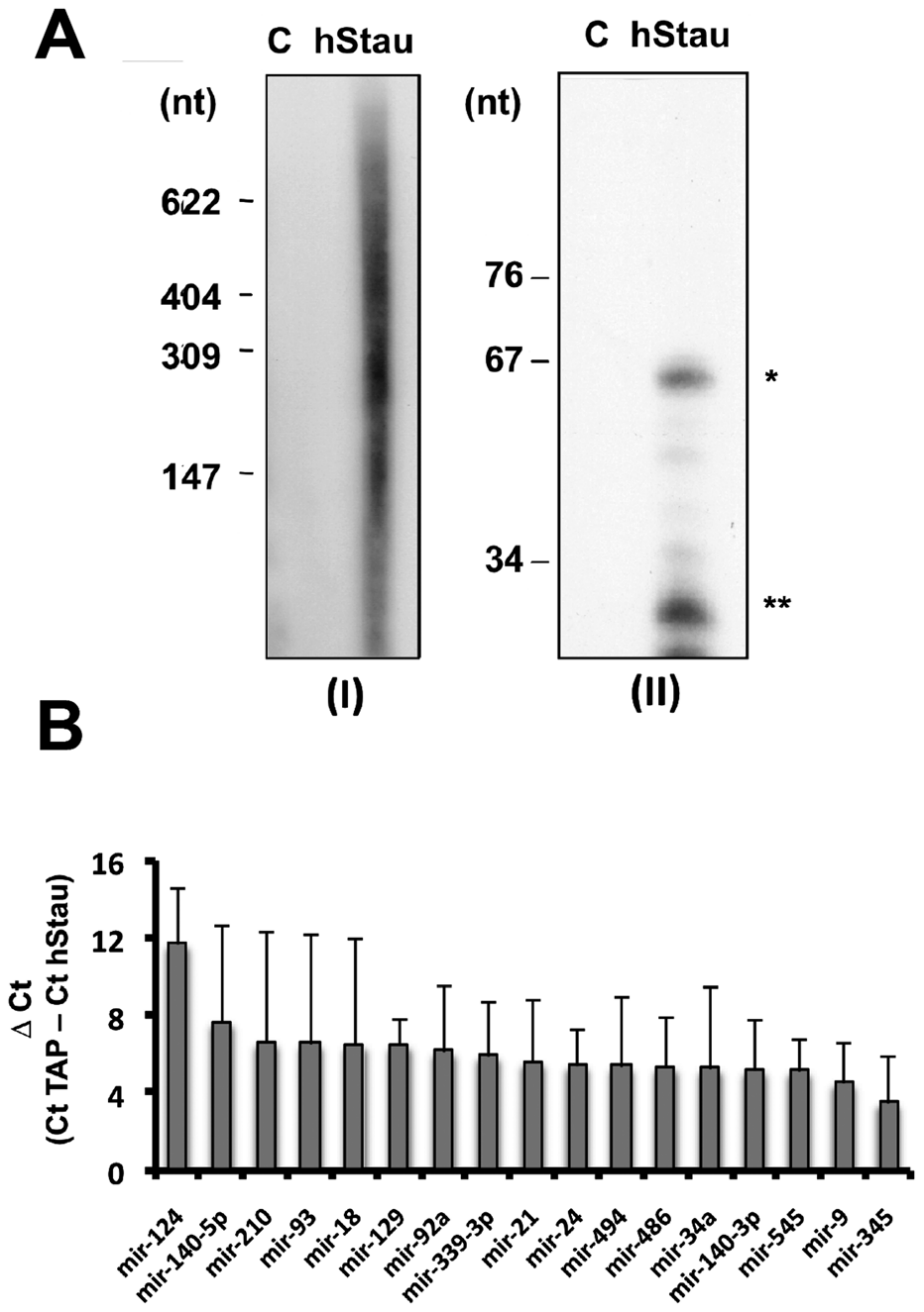
Analysis of hStaufen1-associated RNA. Cultures of HEK293T cells were transfected with pChStaufen1-TAP (hStau) or pC-TAP (C) and soluble extracts were used for TAP purification. (A) The RNA associated was isolated from the purified complexes, 5′-end radiolabeled using γ-^32^P-ATP and the different RNA sizes were analysed in two denatured polyacrylamide gels, 4% (I) and 15% (II). * Indicates the size corresponding to pre-microRNAs. ** Indicates the size corresponding to mature miRNAs. The mobility of molecular weight markers is indicated to the left. (B) Small RNAs were studied using TaqMan RT-qPCR Applied multiplex to analyse 384 miRNAs with specific primers. The differences in Ct values for parallel control and hStau1 complexes are shown. Results are averages and standard deviations of 3 replicate purifications. Under the conditions used, a ΔCt of 3.3 corresponds to a ten-fold difference in RNA concentration.

**Figure 3:**
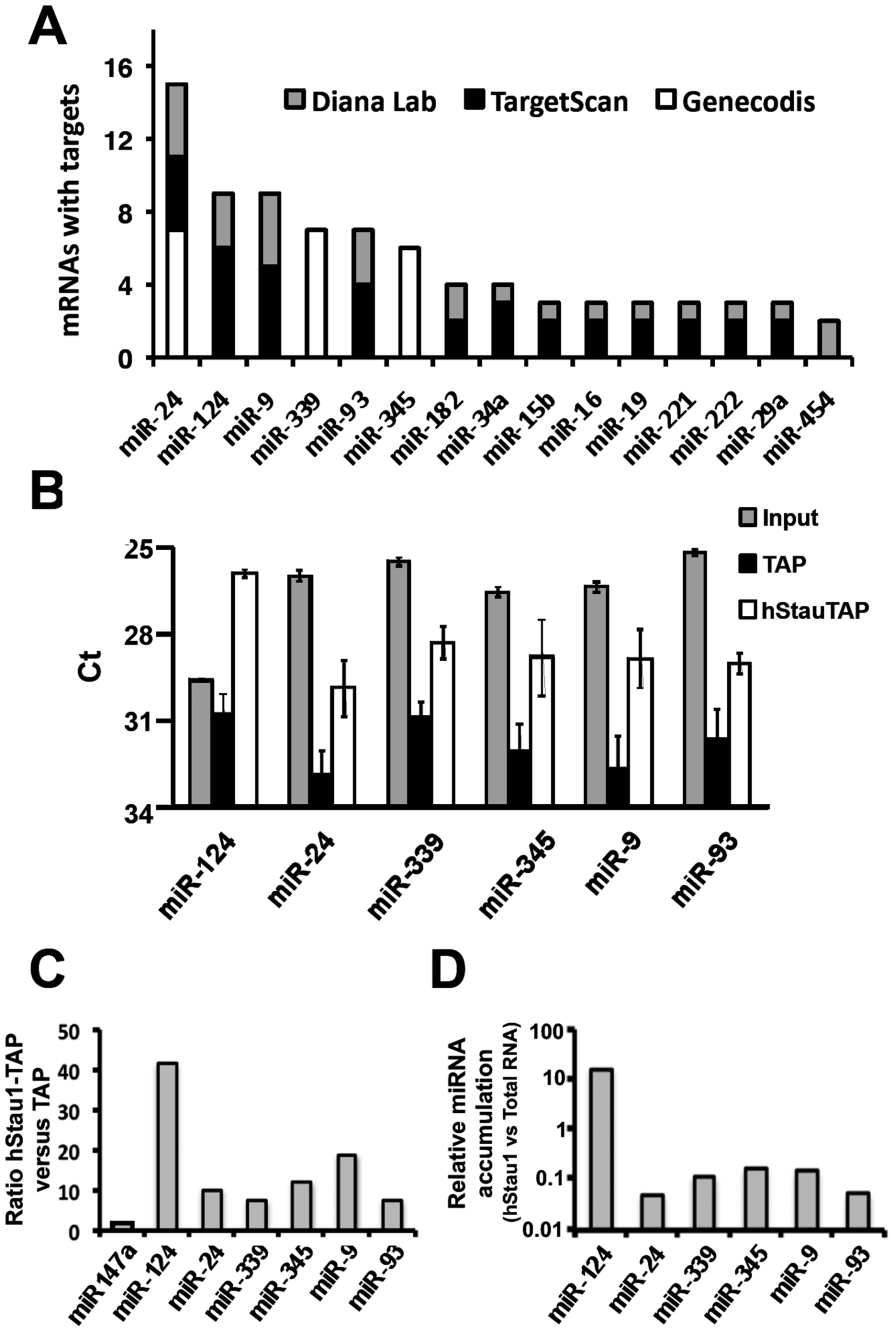
Analysis of the most representative miRNAs associated to the hStau1 complexes. (A) A bioinformatic analysis was performed using 2 prediction algorithms (TargetScan and DianaLab) and 1 annotation database (Genecodis) to identify target miRNAs sites in the 66 hStau1-associated mRNAs detected in the transcriptomic analysis (see Table S1). The graph represents the number of mRNA with targets for each miRNA. (B) The selected miRNAs were analysed individually by TaqMan RT-qPCR in RNAs isolated from hStau1 complexes purified from HEK293T cells transfected with either pChStaufen1-TAP (white bars), pCTAP plasmid (black bars), or from total cell RNA (grey bars). Values are averages and standard deviations of 3 biological replicates. (C) The relative concentrations of each miRNA in hStau1 versus control TAP complexes are represented as comparison with miR-147a used as an example of miRNA not associated to hStau1. (D) The relative concentrations of each miRNA in RNA samples isolated from purified hStau1 complexes or from total cell RNA are represented.

In view of these results we used a similar strategy to test whether cellular miRNAs are present in purified hStau1 complexes and eventually to identify those preferentially associated. The RNA present in purified hStau1 complexes was isolated, dephosphorylated and 5′-labelled with polynucleotide kinase and gamma-^32^P-ATP. Heterogeneous-sized RNAs were detectable in these complexes but not in parallel TAP purifications used as controls (Fig. 2A, left panel). In addition, RNAs with a size compatible with miRNAs and pre-miRNAs were also detected (Fig. 2A, right panel). To identify which miRNAs are present in the complex, the hStau1-associated RNA was used for a RT-qPCR screening that included 384 common human miRNAs, as indicated in Materials and Methods. Although a previous expression analysis indicated that many human miRNAs were not detectable in HEK293T cells by small RNA sequencing [44] more than 65% of those present in the screening chip were clearly detected in total cell samples of these cells (data not shown and Fig. 3 below). Several miRNAs were detected in purified complexes at concentrations much higher (10^1^ to 10^4^ fold) than in control TAP purifications (Fig. 2B). If the associated miRNAs were functionally relevant we would expect to find mRNAs containing specific targets also associated to the hStau1 complexes. To evaluate this possibility we performed a transcriptomic analysis of the RNAs present in the complexes using Affymetrix chips, as indicated in Materials and Methods. Around 1000 transcripts were at least 2-fold enriched in the RNA associated to hStau1 as compared with total cell transcriptome and, among those, 66 transcripts were at least 4-fold enriched (Table S1). These 66 mRNAs preferentially associated to hStau1 were screened for the presence of target sequences specific for the miRNAs shown in Fig. 2B using two informatic tools (Diana Lab and TargetScan) and one database of experimentally validated targets (Genecodis) and the results are presented in Fig. 3A. miRNAs 124, 24, 9, 339, 93 and 345 showed the highest number of mRNAs with target sequences and were chosen for further analysis. To confirm their association to hStau1 complexes, replicate purifications were analysed by miRNA-specific RT-qPCR assays, using TAP purifications and total cell RNA as controls. The results are presented in Fig. 3B and indicated that these miRNAs were clearly detectable in total cell extracts (i.e. the Ct values observed indicated miRNA concentrations 10^2^–10^4^-fold higher than the detection level set at Ct = 37) and all selected miRNAs were associated to hStau1 complexes compared to control TAP complexes (Fig. 3B). Particularly interesting were miR-124 and miR-9, that showed the highest hStau1 vs TAP ratio, using as a control miR-147a, that was not present among those detected in the initial screening (Fig. 3C). In addition, miR-124 was the only miRNA among those tested that showed higher concentration in the hStau1-associated RNA than in total cell RNA (Fig. 3D).

### miR-124 accumulates in human Staufen1 complexes

**Figure 4:**
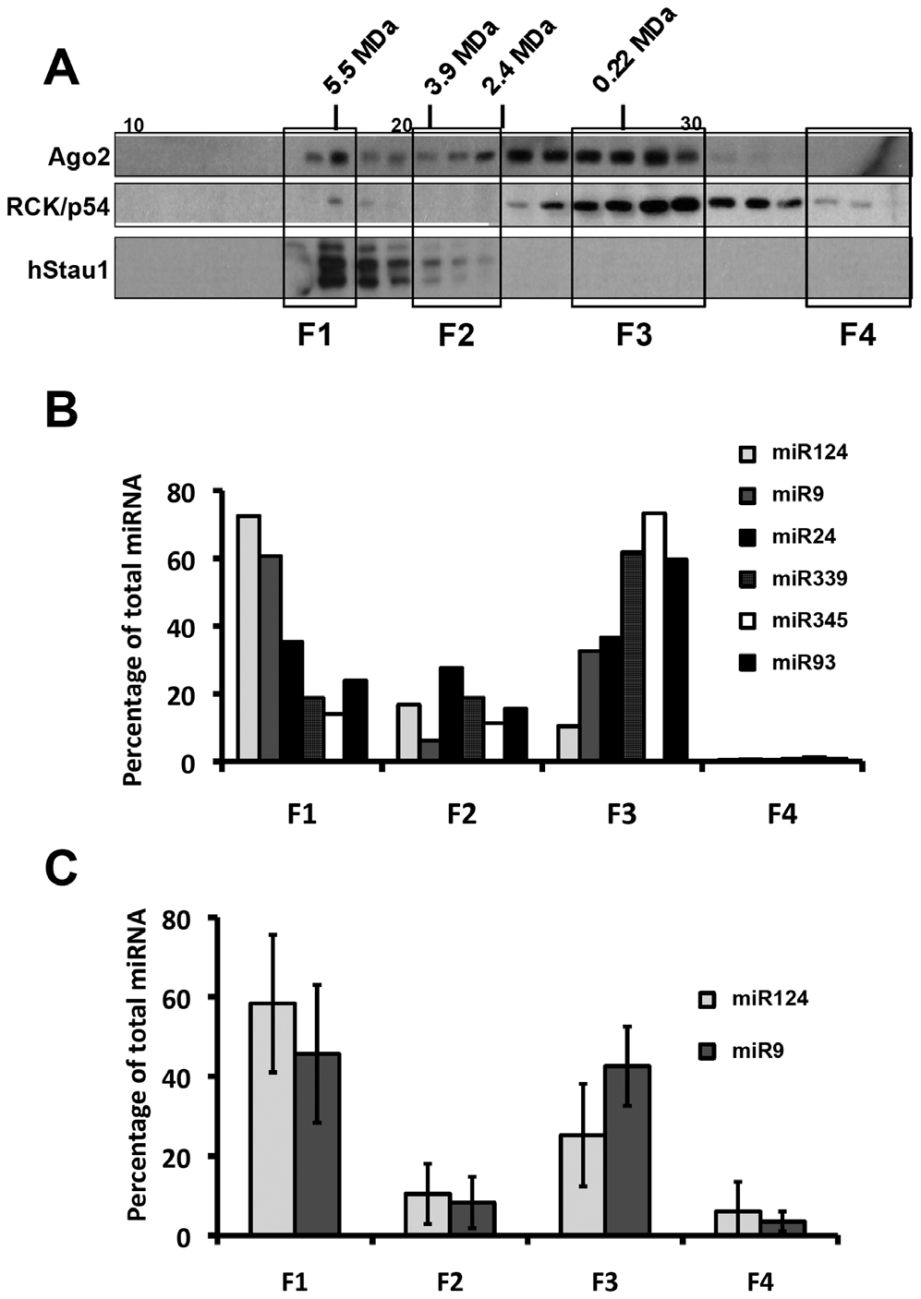
Analysis of hStau1 complexes and the associated miRNAs in human neuroblastoma cells. Soluble extracts derived from the SH-SY5Y neuroblastoma cell line were filtered on a Sephacryl S-400 column. (A) The various fractions were analysed by Western-blot with antibodies specific for Ago2, RCK/p54 and hStau1. (B) The fractions were grouped in 4 pools: F1 (17, 18), F2 (21,22,23), F3 (26,27,28,29) and F4 (34,35,36 as a negative control). RNA was isolated in each pool and analysed by TaqMan RT-qPCR to quantify the six miRNAs most prevalent in hStau1 complexes. (C) The amounts of miR-124 and miR-9 were determined in the fractions pools F1 to F4 derived from 3 independent filtration experiments. Values are averages and standard deviations and represent the amount of miRNA present in each fraction pool as percentage of total miRNA recovered from F1+F2+F3+F4 pools.

The results on miRNA association to hStau1 complexes were obtained in HEK293T cells transfected with a tagged hStau1 protein. To verify these results in a more physiological setting we used human neuroblastoma SH-SY5Y cells, since both miR-124 and miR-9 are highly expressed in neural cells [45], [46]. Total cell extracts were fractionated in a Sephacryl S400 column and the mobility of hStau1-containing complexes was determined by Western-blot. As presented in Fig. 4A, a major peak with a molecular mass>5 MDa was detected, as previously reported [12]. Parallel determination of the mobilities of Ago2 and RCK/p54 markers indicated their distribution in a lower molecular weight region, but a small amount of Ago2 co-migrated with the hStau1-containing complexes (Fig. 4A), in agreement with the co-immunoprecipitation results presented in Fig. 1B. To determine the potential presence of miRNAs in the various size fractions, 4 pools were generated: F1 contained the hStau1 complexes; F3 included most of the Ago2 and RCK/p54 markers; F2 covered sizes intermediate between F1 and F3; F4 was used as a negative control and included low-molecular weight complexes lacking any of the above markers. The distribution of miRNAs among these F1–F4 pools was determined by RT-qPCR and is presented in Fig. 4B. These analyses do not allow to compare the accumulations of the various miRNAs, since the efficiency of each RT-qPCR reaction might be different, but provide information on the distribution of each miRNA between the various size classes. All miRNAs tested were detected in the hStau1-containing F1 pool and, interestingly, miR-124 and miR-9 were preferentially found in this fraction. These results were verified for miR-124 and miR-9 in three independent filtration experiments and the data are presented in Fig. 4C. In addition, miR-124 showed higher concentration in the hStau1 fractions than in the initial cell extract, whereas the rest of the miRNAs analysed were not enriched in the hStau1 complexes (Fig. 3D).

### The size pattern of miR-124-containing complexes changes during neuroblast differentiation

**Figure 5:**
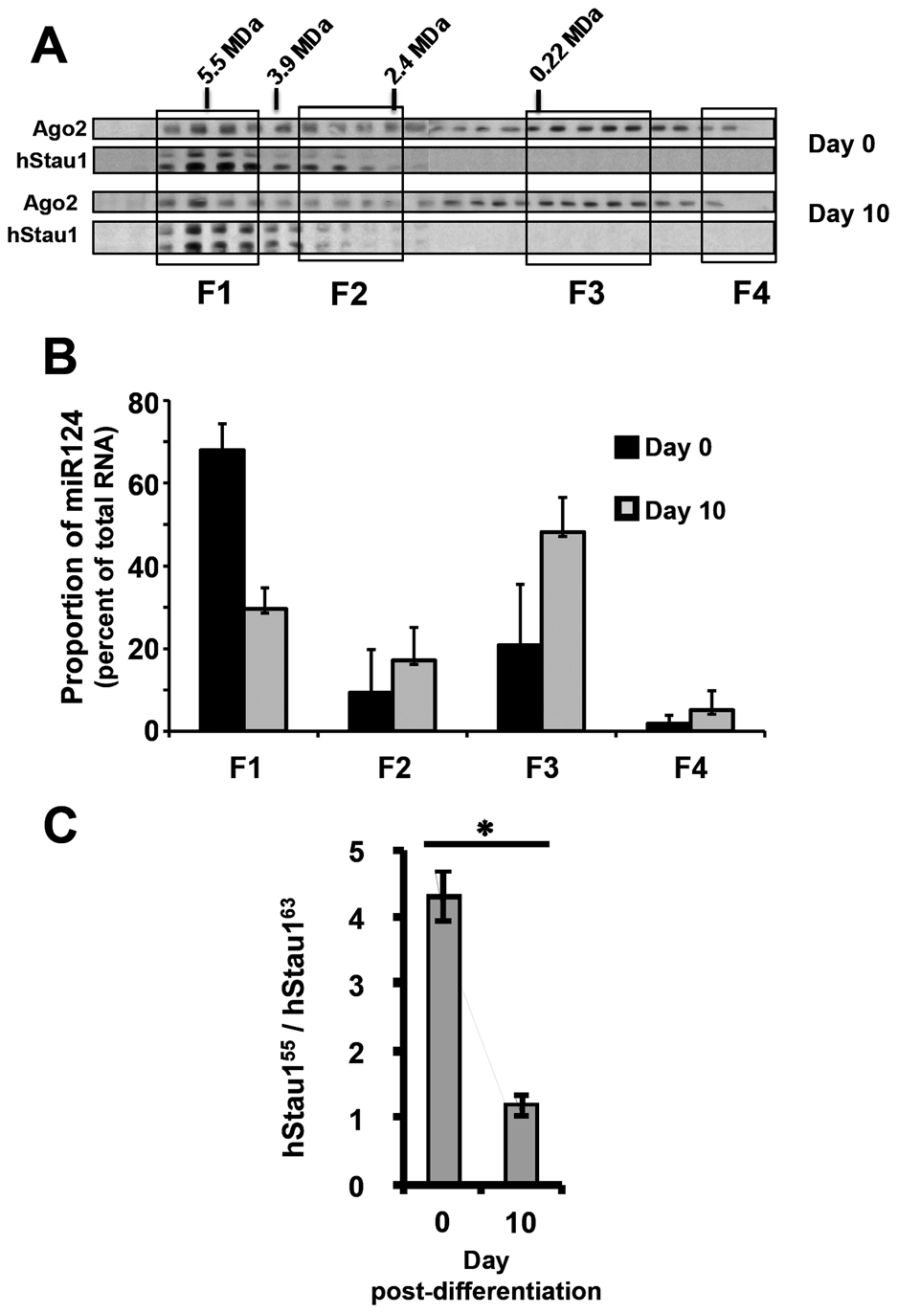
Association of miR-124 to hStau1 complexes in undifferentiated and differentiated neuroblastoma cells. Cultures of SH-SY5Y neuroblastoma cells were differentiated as described in Materials and Methods. Total cell extracts were isolated from cells prior to differentiation (day 0) or at a final stage of differentiation (day 10) and fractionated on a Sephacryl S-400 column. (A) The various fractions were analysed by Western-blot with antibodies specific for Ago2 and hStau1 The gels to analyse hStau1 were run longer than in Fig. 4 to better separate hStau1 55 and 63 kDa isoforms. (B) RNA was isolated from the fraction pools indicated and the concentration of miR-124 was determined by TaqMan RT-qPCR. (**C**) The amounts of hStau1^55^ and hStau1^63^ isoforms were determined by Western-blot (see A) and their ratios is presented as average and standard deviations of 3 determinations. * indicates a p-value<0.05 in a two-tailed Student's t-test.

In agreement with the reported role of miR-124 in neuronal cell differentiation in chick and mouse [47]–[49], a large increase in the total miR-124 concentration was observed in human neuroblastoma SH-SY5Y cells upon differentiation in vitro (Fig. S1). To analyse the size pattern of miR-124–containing complexes during differentiation, total cell extracts derived from SH-SY5Y cells were prepared at days 0 and 10 in the differentiation process and fractionated by gel filtration on Sephacryl S400 as indicated above. The size of the hStau1 complexes did not change upon differentiation (Fig. 5A). Fraction pools F1 to F4 were generated as indicated in Fig. 4 and the RNA was used for miR-124 determinations using RT-qPCR. The results are presented in Fig. 5B and indicated a strong change in its distribution. Whereas most of the miR-124 co-migrated with the hStau1 complexes in undifferentiated cells, it was mostly present in smaller complexes co-migrating with Ago2 when the cells became differentiated. In addition, a large alteration in the ratio of hStau1 isoforms present in the complexes was apparent (Fig. 5A). In undifferentiated SH-SY5Y cells, most of the hStau1 present corresponded to the hStau1^55^ form, but upon in vitro differentiation similar amounts of hStau1^55^ and hStau1^63^ were detected (Fig. 5C).

### Human Staufen1 protein is essential for a normal dendritic arborisation in vitro

**Figure 6:**
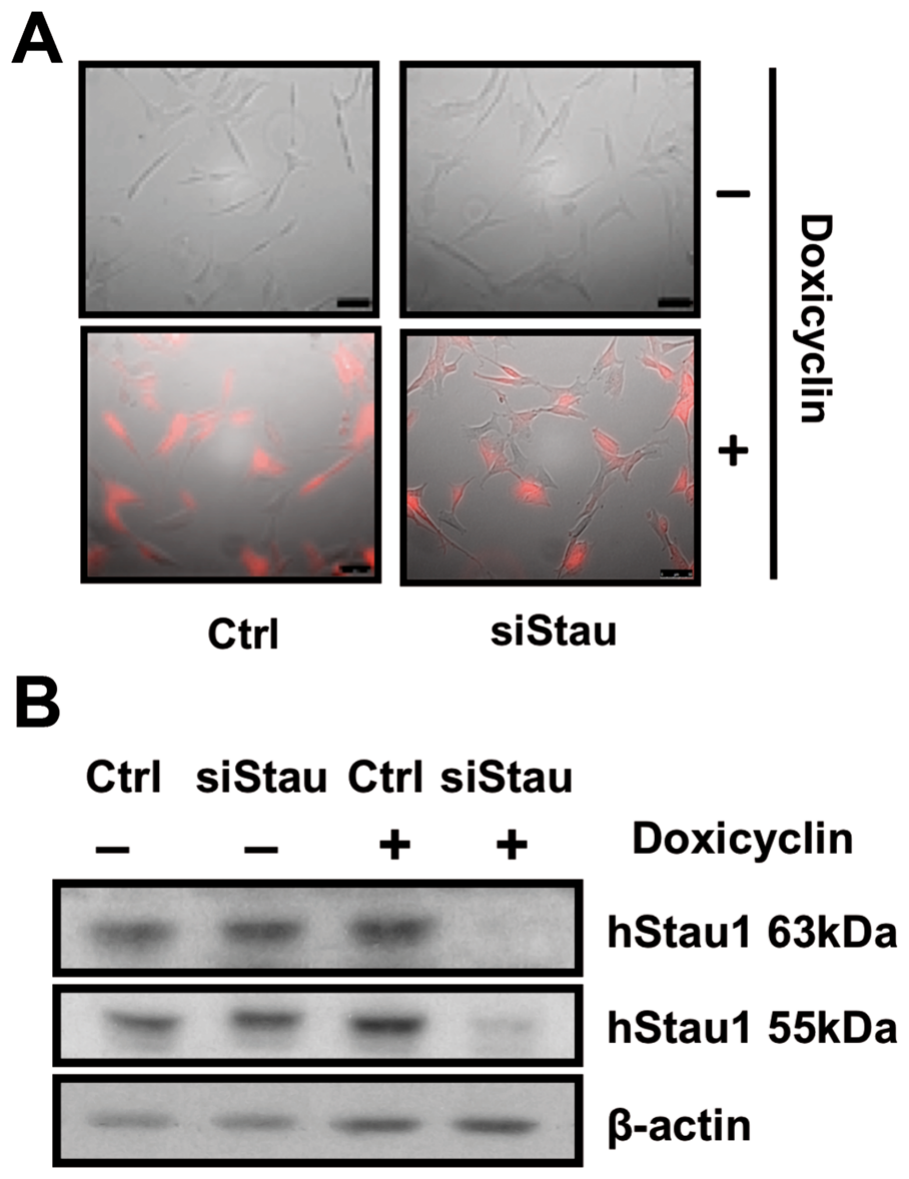
Regulated silencing of hStau1 protein in neuroblastoma cells. Cultures of neuroblastoma SH-SY5Y cells were transduced with a lentiviral construct able to express, under a doxicyclin inducible promoter, RFP and an shRNA with a hStau1 specific silencer (siStau) or an empty shRNA as a control (Ctrl). (A) Combined phase-contrast and fluorescence images of cells treated (bottom) or untreated (top) with doxicyclin. Bar scale correspond to 10 µm. (B) Cell extracts were obtained from the cultures described above and analysed by Western-blot using antibodies specific for hStau1 or ß-actin.

The specific association of miR-124 with hStau1 complexes and the alterations observed in this interaction during human neuroblast differentiation prompted us to address the role of hStau1 in this process. To this aim we generated cell lines derived from the SH-SY5Y neuroblastoma in which the hStau1 gene could be silenced in a regulated fashion. Lentiviral constructs were produced using the pTRIPZ plasmid as a vector in which hStau1-specific silencing sequences were inserted within the backbone of miR-30. The artificial silencing miRNA is expressed by a minimal CMV promoter under the regulation of the Tet operator/Tet repressor in a bicistronic mRNA also encoding the red fluorescent protein (RFP) as a marker (see Fig. S2 for a diagram). Lentiviral particles generated with these plasmids were used to transduce SH-SY5Y cells and the expression of hStau1-specific siRNAs and RFP was induced by addition of doxicyclin. As presented in Fig. 6A, addition of the antibiotic induced the expression of RFP in cells transduced with the siStau lentivirus (siStau) as well as in control cells transduced with lentiviruses generated with empty pTRIPZ plasmid (Ctrl). However, Western-blot analysis of hStau1 accumulation indicated that the hStau1 gene was silenced only in cells transduced with siStau lentivirus and only upon addition of doxicyclin (Fig. 6B).

**Figure 7:**
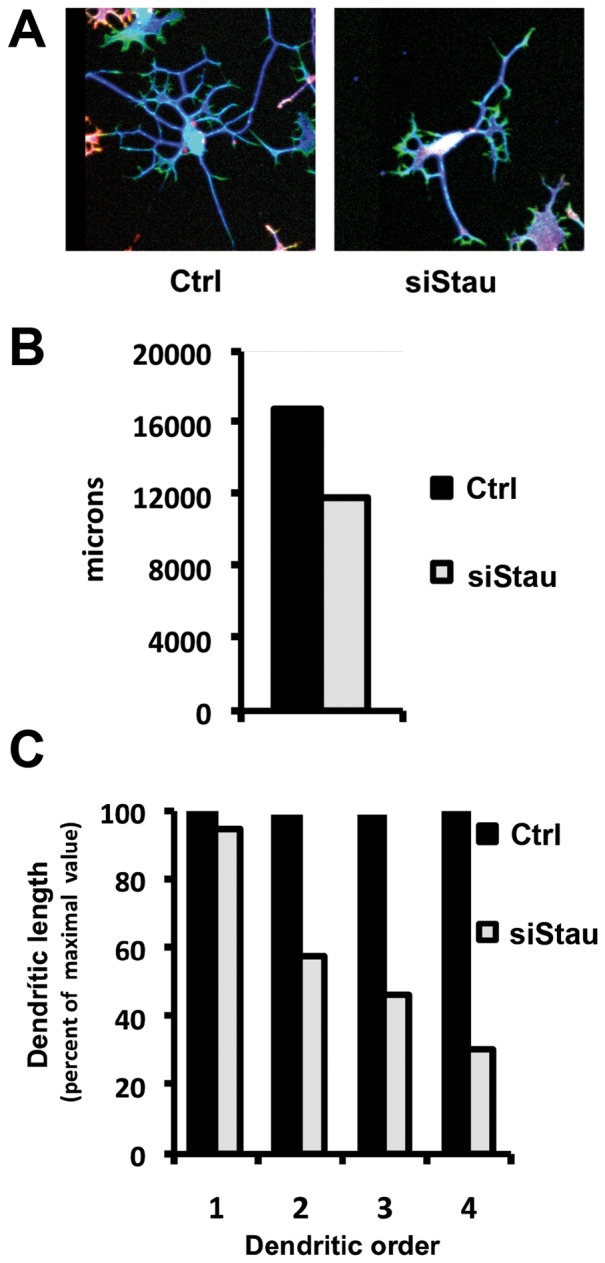
Morphology of differentiated neuroblastoma cells silenced for hStau1 gene. Cultures of SH-SY5Y neuroblastoma cells previously transduced with control (Ctrl) or hStau1-specific (siStau) silencing lentiviruses were treated for 72 h with doxicyclin and then induced for differentiation as described in Materials and Methods. At day 7 post-differentiation, the cultures were fixed and immunostained with an antibody specific for ßIII-tubulin (blue). Phalloidin (green) were used to detect actin filaments. Red colour corresponds to the RFP signal. Dendrites of 50 cells chosen at random were measured for each sample. (A) Representative images of not silenced and silenced differentiated SH-SY5Y cells. (B) The graph shows the total dendrite length for control or silenced cells. (C) The graph presents the dendritic length per dendritic order.

**Figure 8:**
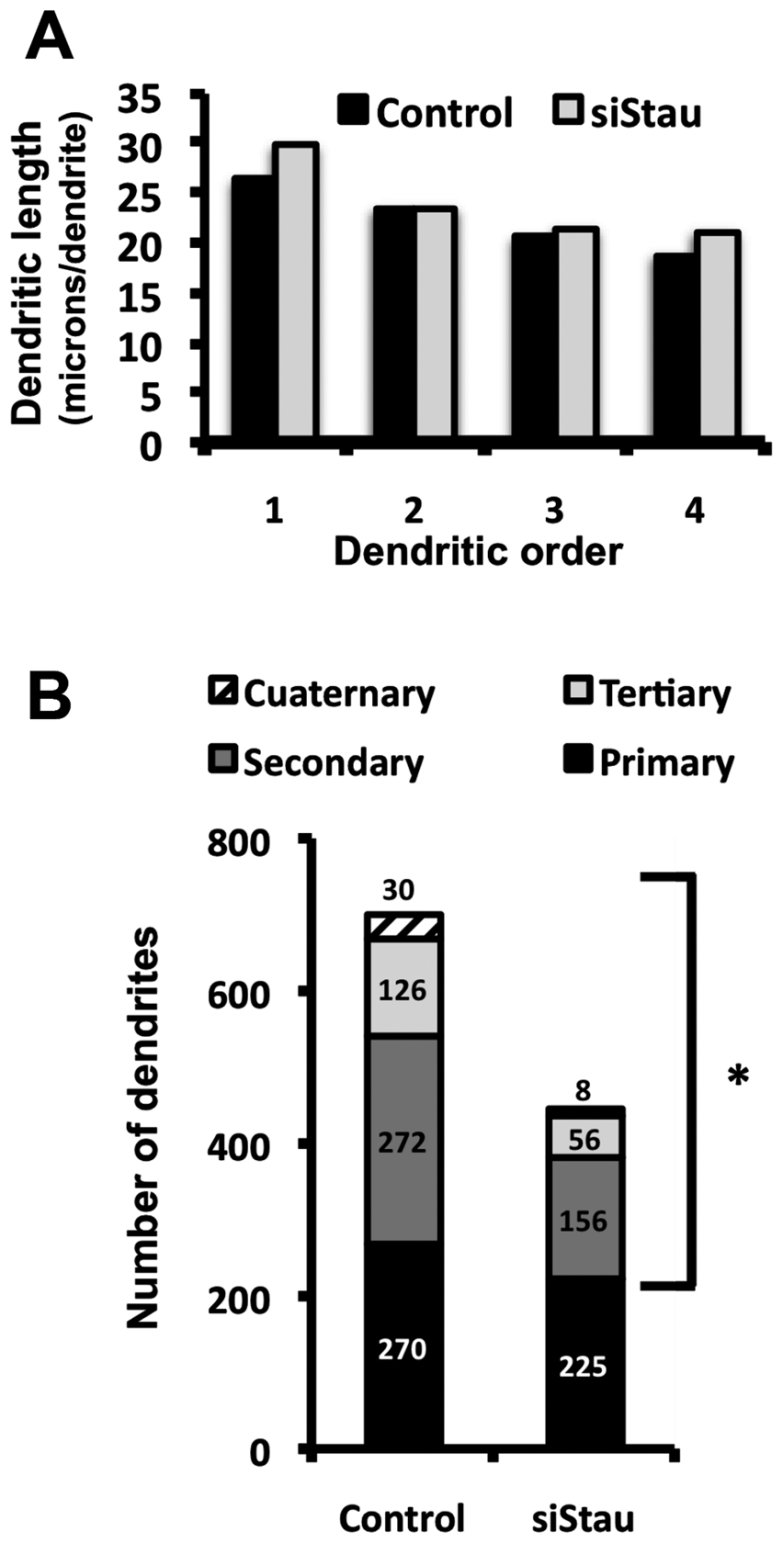
Number and length of dendrites in differentiated hStau1-silenced neuroblastoma cells. The length of dendrites from 50 hStau1-silenced or control differentiated cells was measured. (A) The graph represents the average length per dendrite for each dendritic order. (B) Quantification of the total dendrite number for each dendritic order. The statistical significance was determined using a two-tailed Student's t-test after verifying that each value series conform to a Normal distribution using the Shapiro-Wilk normality test. * indicates a p-value<0.05.

Once the conditions for regulated silencing of hStau1 were established, we set out to test whether it was required for neuroblast differentiation or for the maintenance of the differentiated state in vitro. Cultures of SH-SY5Y cells transduced with either control or siStau lentiviruses were treated with doxicyclin for 3 days until the level of hStau1 protein reached a minimum. Then differentiation *in vitro* was induced as indicated in Materials and Methods and the phenotype of the cells was studied after staining of ß3-tubulin and actin. Silencing of hStau1 did not alter the capacity of undifferentiated neuroblast to replicate in vitro (Fig. S3A) and did not change the proportion of neuroblasts that differentiated to a neuron-like phenotype (Fig. S3B,C). However, a detailed analysis of the structure of neuron-like differentiated cells revealed a clear alteration in the dendrite organisation of hStau1-silenced versus control cells (Fig. 7A). The total dendritic length was smaller in the former, although the difference was not statistically significant (Fig. 7B). On the contrary, when the results were analysed after classification by dendritic order, significant differences were observed in the total dendritic length of secondary to quaternary dendrites (Fig. 7C). These results could be the consequence of a reduction of the number of dendrites or a decrease in the dendritic length. Analysis of the length per dendrite excluded a reduction in their size (Fig. 8A) but a statistically significant reduction in the number of secondary to quaternary dendrites was apparent (Fig. 8B).

To test whether hStau1 is relevant in the maintenance of the differentiation state SH-SY5Y cells were induced to differentiate as indicated in Materials and Methods and, at day 5 after induction, doxicyclin was added to the media to induce hStau1 silencing. After 2 days, when arborisation was almost completed, the level of expression of hStau1 was reduced but no differences were apparent in the phenotype of the neuron-like cells (Fig. S4; compare to Fig. 7A).

## Discussion

The development of the mammalian nervous system, including the establishment of correct neuronal connections and specific synapses, is a daunting task that can only be achieved by the properly regulated activities, in time and space, of a proteome that is highly diversified as compared to the encoding genome (for a recent review see [50]. Thus, multiple layers of regulation amplify the capacity of the mammalian genome to meet the developmental and functional requirements of the nervous system, including transcriptional control, regulation of alternative splicing, mRNA localisation and spatio-temporal control of specific mRNA translation by RNA-binding proteins and miRNAs [51]–[53]. The latter steps in the control of gene expression require the action of specific proteins that are present in RNA-containing granules and mediate the localisation and regulated translation of the specific mRNAs included [51], [54]. Here we identify miR-124 and miR-9 as miRNAs specifically associated to hStau1, one of these proteins, and show that hStau1 is important for the proper differentiation of human neuroblastoma to neuron-like cells.

Consistent with the identification of specific miRNAs associated to purified hStau1 complexes (Figs. 2, 3), essential elements of the miRNA-mediated silencing machinery, particularly Ago2 protein, were found among the factors present in these complexes (Fig. 1), as well as a number of mRNAs that are potential targets for these miRNAs (Fig. 3A; Table S1). All together, these findings suggest that, at least in part, the purified hStau1 complexes might represent transport RNA granules in which specific mRNAs are translationally repressed by miRNA-mediated silencing, i.e. the association of the RISC complex to the hStau1-containing granules might be indirect and reflect that these granules contain mRNAs containing specific miRNA target sites allowing transient silencing. This is not unprecedented, as FMRP-containing RNA complexes also are associated to RISC elements [55], [56]. Interestingly, two of the most prominent miRNAs found associated to hStau1 during screening were miR-124 and miR-9, which have been described as highly relevant for neural development [24], [47]–[49]. Although screening was carried out in the HEK293T cell line, this finding is not surprising, as this cell line has been shown to share many properties with neural cells [43]. Furthermore, the association of both miR-124 and miR-9 to hStau1 complexes was verified in non-transfected SH-SY5Y human neuroblastoma cells (Figs. 4, 5). Further studies will be needed to extend these observations to other neuroblastoma cell lines or primary cells.

Previous reports have documented that miR-124 participates in the neural development by modifying several of the regulation layers indicated above, like transcription [24], alternative splicing [48] or specific protein silencing [49]. Here we show that, as expected, the levels of miR-124 increase during the differentiation of neuroblastoma to neuron-like cells in vitro (Fig. S1) and, furthermore, its pattern of association to hStau1 complexes changes along this process (Fig. 5), suggesting a role for hStau1 in neural differentiation. Early work in *D. melanogaster* documented that dmStaufen is important for neuronal precursor cell fate [5] and mammalian Staufen1 has been shown to contribute to spine morphology, synaptic function and long-term potentiation in rats [57], [58] and mice [59], but a function of hStau1 in earlier stages of neurite development has not been tested. Here we developed a regulated hStau1 silencing strategy (Fig. S2; Fig. 6) to analyse its role in neuroblast differentiation in vitro and show that it is important for dendrite outgrow but not for the maintenance of the neuron-like morphology after differentiation (Figs. 7, 8; Fig. S4). Our results are consistent with those reported by Vessey et al. for a RNA-binding mutant of mStau1 [59] but the phenotype reported here by silencing is stronger, suggesting that other regions of hStau1 protein in addition to dsRNA binding domain 3 are relevant for its function during neural development.

In this report we present two independent but related observations: (i) Essential elements of the gene silencing system are associated to hStau1 protein in human cells and specific miRNAs are present in the RNA-containing complexes containing hStau1. Among these, miR-124 stands out as particularly enriched in hStau1-containing complexes and is over-expressed upon differentiation of human neuroblastoma cells in vitro and (ii) Expression of hStau1 is essential for proper dendritic arborisation during neuroblastoma cell differentiation, yet it is not necessary for maintenance of the differentiated state. Taken together, these results suggest that one or several specific mRNA targets of hStau1 might be responsible for neuron arborisation. Screening of the hStau1-associated mRNAs identified by microarray hybridisation (Table S1) did not yield any reasonable gene candidate and therefore we used data from a more extensive analysis recently performed by deep-sequencing of RNAs specifically recognised by hStau1 [60]. Three hStau1-specific mRNAs contained predicted miR-124 targets and have been described as related to neuron function: The homeobox-containing gene *engrailed2* (*en2*), which is involved in autism disorder [61], [62], the *magnesium transporter 1* gene (*magt1*), identified by differential expression during epilepsy [63] and most interestingly, *synaptic cell-adhesion molecule2*/*leucine-rich repeat and fibronectin III domain-containing molecule1* (*salm2/lrfn1*) gene. The latter is a member of the SALM/lrfn family of adhesion molecules that has been shown to play a role in dendritic arborisation [64], [65]. Thus, over expression of SALM2/lrfn1 protein led to increased number of branches but no significant changes in process length [65], a phenotype identical to that described here (Fig. 8). Therefore, it is tempting to speculate that the defects in neuron arborisation induced by hStau1 silencing might be due, at least in part, by the lack of proper mRNA localisation and expression of *SALM2/lrfn1* gene. Further studies will be needed to experimentally test this proposal.

## Supporting Information

Figure S1 **Induction of miR-124 upon neuroblast differentiation.** Cultures of SH-SY5Y neuroblastoma cells were differentiated as described in Materials and Methods. Total cell extracts were isolated from cells prior to differentiation (day 0) or at a final stage of differentiation (day 10). Total cell RNA was isolated and the concentration of miR-124 was determined by TaqMan RT-qPCR.
(TIF)

Figure S2 **Experimental strategy for the regulated silencing of hStau1.** Specific silencing sequences were inserted into the miR-30 skeleton present in pTRIPZ plasmid. The recombinant miRNA is expressed from a minimal CMV promoter under the control of Tet repressor, in a bicistronic mRNA also containing the RFP as a marker. The construct was used to generate lentiviral particles containing VSV G glycoprotein. Target cells were transduced with the recombinant lentivirus and selected with puromycin. Induction with doxicyclin leads to the expression of RFP and the hStau1 silencing RNA.
(TIF)

Figure S3 **General properties of hStau1-silenced neuroblastoma cells.** Cultures of SH-SY5Y neuroblastoma cells were transduced with control (black bars) or hStau1 silencing lentiviruses (grey bars). (A) The cells were cultivated and the number of cells counted. The relative number of cells at time 0 and 2 days after incubation are presented. (B) The cultures were induced for differentiation as indicated in Materials and Methods and stained to reveal ß3-tubulin and actin at day 7. A representative field of a differentiated culture is presented to show the various cell phenotypes obtained: Fibroblast-like (1; green), Intermediate (2; grey) and neuron-like (3; blue). (C) The number of cells showing the various phenotypes were counted in control (black bars) or hStau1-silenced (grey bars) cultures and is presented as percent of total cells.
(TIF)

Figure S4 **Morphology of differentiated neuroblastoma cells upon hStau1 silencing.** Cultures of SH-SY5Y neuroblastoma cells previously transduced with control (Ctrl) or hStau1-specific (siStau) silencing lentiviruses were induced for differentiation as described in Materials and Methods. Starting at day 5 post-differentiation, cells were treated with doxicyclin during 5 days. From day 7 post-differentiation hStau1 expression levels were reduced. At 10 days post-differentiation the cultures were fixed and immunostained with antibodies specific for ßIII-tubulin (blue) and phalloidin (green). Red colour corresponds to the RFP signal derived from the lentiviral constructs. The images show 2 representative fields of Ctrl- or siStau-transduced cultures.
(TIF)

Table S1 **List of mRNAs associated to hStau1.**
(DOC)

## References

[1] Bassell GJ, Kelic S (2004) Binding proteins for mRNA localization and local translation, and their dysfunction in genetic neurological disease. Curr Opin Neurobiol 14:574–581. 15464890 10.1016/j.conb.2004.08.010

[2] Bassell GJ, Oleynikov Y, Singer RH (1999) The travels of mRNAs through all cells large and small. Faseb J 13:447–454. 10064611 10.1096/fasebj.13.3.447

[3] Knowles RB, Sabry JH, Martone ME, Deerinck TJ, Ellisman MH, et al (1996) Translocation of RNA granules in living neurons. J Neurosci 16:7812–7820. 8987809 10.1523/JNEUROSCI.16-24-07812.1996 PMC6579227

[4] St Johnston D, Beuchle D, Nusslein-Volhard C (1991) Staufen, a gene required to localize maternal RNAs in the Drosophila egg. Cell 66:51–63. 1712672 10.1016/0092-8674(91)90138-o

[5] Broadus J, Fuerstenberg S, Doe CQ (1998) Staufen-dependent localization of prospero mRNA contributes to neuroblast daughter-cell fate. Nature 391:792–795. 9486649 10.1038/35861

[6] Ferrari F, Mercaldo V, Piccoli G, Sala C, Cannata S, et al (2007) The fragile X mental retardation protein-RNP granules show an mGluR-dependent localization in the post-synaptic spines. Mol Cell Neurosci 34:343–354. 17254795 10.1016/j.mcn.2006.11.015

[7] Kim M, Ceman S (2012) Fragile X mental retardation protein: past, present and future. Curr Protein Pept Sci 13:358–371. 22708486 10.2174/138920312801619420

[8] Zalfa F, Giorgi M, Primerano B, Moro A, Di Penta A, et al (2003) The fragile X syndrome protein FMRP associates with BC1 RNA and regulates the translation of specific mRNAs at synapses. Cell 112:317–327. 12581522 10.1016/s0092-8674(03)00079-5

[9] Kohrmann M, Luo M, Kaether C, DesGroseillers L, Dotti CG, et al (1999) Microtubule-dependent recruitment of Staufen-green fluorescent protein into large RNA-containing granules and subsequent dendritic transport in living hippocampal neurons. Mol Biol Cell 10:2945–2953. 10473638 10.1091/mbc.10.9.2945 PMC25535

[10] Marión RM, Fortes P, Beloso A, Dotti C, Ortín J (1999) A human sequence homologue of staufen is an RNA-binding protein that localizes to the polysomes of the rough endoplasmic reticulum. Mol Cell Biol 19:2212–2219. 10022908 10.1128/mcb.19.3.2212 PMC84014

[11] Wickham L, Duchaine T, Luo M, Nabi IR, DesGroseillers L (1999) Mammalian staufen is a double-stranded-RNA- and tubulin-binding protein which localizes to the rough endoplasmic reticulum. Mol Cell Biol 19:2220–2230. 10022909 10.1128/mcb.19.3.2220 PMC84015

[12] Villacé P, Marión RM, Ortín J (2004) The composition of Staufen-containing RNA granules from human cells indicate a role in the regulated transport and translation of messenger RNAs. Nucleic Acids Res 32:2411–2420. 15121898 10.1093/nar/gkh552 PMC419443

[13] Krichevsky AM, Kosik KS (2001) Neuronal RNA granules: a link between RNA localization and stimulation-dependent translation. Neuron 32:683–696. 11719208 10.1016/s0896-6273(01)00508-6

[14] Barbee SA, Estes PS, Cziko AM, Hillebrand J, Luedeman RA, et al (2006) Staufen- and FMRP-containing neuronal RNPs are structurally and functionally related to somatic P bodies. Neuron 52:997–1009. 17178403 10.1016/j.neuron.2006.10.028 PMC1955741

[15] Carmell MA, Xuan Z, Zhang MQ, Hannon GJ (2002) The Argonaute family: tentacles that reach into RNAi, developmental control, stem cell maintenance, and tumorigenesis. Genes Dev 16:2733–2742. 12414724 10.1101/gad.1026102

[16] Meister G, Landthaler M, Patkaniowska A, Dorsett Y, Teng G, et al (2004) Human Argonaute2 mediates RNA cleavage targeted by miRNAs and siRNAs. Mol Cell 15:185–197. 15260970 10.1016/j.molcel.2004.07.007

[17] Cai X, Hagedorn CH, Cullen BR (2004) Human microRNAs are processed from capped, polyadenylated transcripts that can also function as mRNAs. Rna 10:1957–1966. 15525708 10.1261/rna.7135204 PMC1370684

[18] Lee Y, Ahn C, Han J, Choi H, Kim J, et al (2003) The nuclear RNase III Drosha initiates microRNA processing. Nature 425:415–419. 14508493 10.1038/nature01957

[19] Eulalio A, Huntzinger E, Izaurralde E (2008) Getting to the root of miRNA-mediated gene silencing. Cell 132:9–14. 18191211 10.1016/j.cell.2007.12.024

[20] Fabian MR, Sonenberg N (2012) The mechanics of miRNA-mediated gene silencing: a look under the hood of miRISC. Nat Struct Mol Biol 19:586–593. 22664986 10.1038/nsmb.2296

[21] Li J, Wan Y, Ji Q, Fang Y, Wu Y (2013) The role of microRNAs in B-cell development and function. Cell Mol Immunol 10:107–112. 23314697 10.1038/cmi.2012.62 PMC4003047

[22] Farazi TA, Spitzer JI, Morozov P, Tuschl T (2011) miRNAs in human cancer. J Pathol 223:102–115. 21125669 10.1002/path.2806 PMC3069496

[23] Lages E, Ipas H, Guttin A, Nesr H, Berger F, et al (2012) MicroRNAs: molecular features and role in cancer. Front Biosci 17:2508–2540. 10.2741/4068 PMC3815439 22652795

[24] Yoo AS, Staahl BT, Chen L, Crabtree GR (2009) MicroRNA-mediated switching of chromatin-remodelling complexes in neural development. Nature 460:642–646. 19561591 10.1038/nature08139 PMC2921580

[25] de Lucas S, Peredo J, Marion RM, Sanchez C, Ortin J (2010) Human Staufen1 protein interacts with influenza virus ribonucleoproteins and is required for efficient virus multiplication. J Virol 84:7603–7612. 20504931 10.1128/JVI.00504-10 PMC2897607

[26] DuBridge RB, Tang P, Hsia HC, Leong PM, Miller JH, et al (1987) Analysis of mutation in human cells by using an Epstein-Barr virus shuttle system. Mol Cell Biol 7:379–387. 3031469 10.1128/mcb.7.1.379-387.1987 PMC365079

[27] Coloma R, Valpuesta JM, Arranz R, Carrascosa JL, Ortin J, et al (2009) The structure of a biologically active influenza virus ribonucleoprotein complex. PLoS Pathog 5:e1000491. 19557158 10.1371/journal.ppat.1000491 PMC2695768

[28] Gimenez-Cassina A, Lim F, Diaz-Nido J (2006) Differentiation of a human neuroblastoma into neuron-like cells increases their susceptibility to transduction by herpesviral vectors. J Neurosci Res 84:755–767. 16802347 10.1002/jnr.20976

[29] Ortín J, Nájera R, López C, Dávila M, Domingo E (1980) Genetic variability of Hong Kong (H3N2) influenza viruses: spontaneous mutations and their location in the viral genome. Gene 11:319–331. 6783473 10.1016/0378-1119(80)90072-4

[30] Wigler M, Pellicer A, Silverstein S, Axel R, Urlaub G, et al (1979) DNA-mediated transfer of the adenine phosphoribosyltransferase locus into mammalian cells. Proc Natl Acad Sci U S A 76:1373–1376. 286319 10.1073/pnas.76.3.1373 PMC383253

[31] Wettenhall JM, Simpson KM, Satterley K, Smyth GK (2006) affylmGUI: a graphical user interface for linear modeling of single channel microarray data. Bioinformatics 22:897–899. 16455752 10.1093/bioinformatics/btl025

[32] Irizarry RA, Hobbs B, Collin F, Beazer-Barclay YD, Antonellis KJ, et al (2003) Exploration, normalization, and summaries of high density oligonucleotide array probe level data. Biostatistics 4:249–264. 12925520 10.1093/biostatistics/4.2.249

[33] Reiner A, Yekutieli D, Benjamini Y (2003) Identifying differentially expressed genes using false discovery rate controlling procedures. Bioinformatics 19:368–375. 12584122 10.1093/bioinformatics/btf877

[34] Meijering E, Jacob M, Sarria JC, Steiner P, Hirling H, et al (2004) Design and validation of a tool for neurite tracing and analysis in fluorescence microscopy images. Cytometry A 58:167–176. 15057970 10.1002/cyto.a.20022

[35] Carmona-Saez P, Chagoyen M, Tirado F, Carazo JM, Pascual-Montano A (2007) GENECODIS: a web-based tool for finding significant concurrent annotations in gene lists. Genome Biol 8:R3. 17204154 10.1186/gb-2007-8-1-r3 PMC1839127

[36] Nogales-Cadenas R, Carmona-Saez P, Vazquez M, Vicente C, Yang X, et al (2009) GeneCodis: interpreting gene lists through enrichment analysis and integration of diverse biological information. Nucleic Acids Res 37:W317–322. 19465387 10.1093/nar/gkp416 PMC2703901

[37] Tabas-Madrid D, Nogales-Cadenas R, Pascual-Montano A (2012) GeneCodis3: a non-redundant and modular enrichment analysis tool for functional genomics. Nucleic Acids Res 40:W478–483. 22573175 10.1093/nar/gks402 PMC3394297

[38] Grimson A, Farh KK, Johnston WK, Garrett-Engele P, Lim LP, et al (2007) MicroRNA targeting specificity in mammals: determinants beyond seed pairing. Mol Cell 27:91–105. 17612493 10.1016/j.molcel.2007.06.017 PMC3800283

[39] Maragkakis M, Alexiou P, Papadopoulos GL, Reczko M, Dalamagas T, et al (2009) Accurate microRNA target prediction correlates with protein repression levels. BMC Bioinformatics 10:295. 19765283 10.1186/1471-2105-10-295 PMC2752464

[40] Maragkakis M, Reczko M, Simossis VA, Alexiou P, Papadopoulos GL, et al (2009) DIANA-microT web server: elucidating microRNA functions through target prediction. Nucleic Acids Res 37:W273–276. 19406924 10.1093/nar/gkp292 PMC2703977

[41] Graham FL, Smiley J, Russell WC, Nairn R (1977) Characteristics of a human cell line transformed by DNA from human adenovirus type 5. J Gen Virol 36:59–74. 886304 10.1099/0022-1317-36-1-59

[42] Campbell SA, Lin J, Dobrikova EY, Gromeier M (2005) Genetic determinants of cell type-specific poliovirus propagation in HEK 293 cells. J Virol 79:6281–6290. 15858012 10.1128/JVI.79.10.6281-6290.2005 PMC1091735

[43] Shaw G, Morse S, Ararat M, Graham FL (2002) Preferential transformation of human neuronal cells by human adenoviruses and the origin of HEK 293 cells. Faseb J 16:869–871. 11967234 10.1096/fj.01-0995fje

[44] Landgraf P, Rusu M, Sheridan R, Sewer A, Iovino N, et al (2007) A mammalian microRNA expression atlas based on small RNA library sequencing. Cell 129:1401–1414. 17604727 10.1016/j.cell.2007.04.040 PMC2681231

[45] Krichevsky AM, King KS, Donahue CP, Khrapko K, Kosik KS (2003) A microRNA array reveals extensive regulation of microRNAs during brain development. Rna 9:1274–1281. 13130141 10.1261/rna.5980303 PMC1370491

[46] Smirnova L, Grafe A, Seiler A, Schumacher S, Nitsch R, et al (2005) Regulation of miRNA expression during neural cell specification. Eur J Neurosci 21:1469–1477. 15845075 10.1111/j.1460-9568.2005.03978.x

[47] Cheng LC, Pastrana E, Tavazoie M, Doetsch F (2009) miR-124 regulates adult neurogenesis in the subventricular zone stem cell niche. Nat Neurosci 12:399–408. 19287386 10.1038/nn.2294 PMC2766245

[48] Makeyev EV, Zhang J, Carrasco MA, Maniatis T (2007) The MicroRNA miR-124 promotes neuronal differentiation by triggering brain-specific alternative pre-mRNA splicing. Mol Cell 27:435–448. 17679093 10.1016/j.molcel.2007.07.015 PMC3139456

[49] Visvanathan J, Lee S, Lee B, Lee JW, Lee SK (2007) The microRNA miR-124 antagonizes the anti-neural REST/SCP1 pathway during embryonic CNS development. Genes Dev 21:744–749. 17403776 10.1101/gad.1519107 PMC1838526

[50] Loya CM, Van Vactor D, Fulga TA (2010) Understanding neuronal connectivity through the post-transcriptional toolkit. Genes Dev 24:625–635. 20360381 10.1101/gad.1907710 PMC2849119

[51] Bramham CR, Wells DG (2007) Dendritic mRNA: transport, translation and function. Nat Rev Neurosci 8:776–789. 17848965 10.1038/nrn2150

[52] Hattori D, Chen Y, Matthews BJ, Salwinski L, Sabatti C, et al (2009) Robust discrimination between self and non-self neurites requires thousands of Dscam1 isoforms. Nature 461:644–648. 19794492 10.1038/nature08431 PMC2836808

[53] Sun AX, Crabtree GR, Yoo AS (2013) MicroRNAs: regulators of neuronal fate. Curr Opin Cell Biol 25:215–221. 23374323 10.1016/j.ceb.2012.12.007 PMC3836262

[54] Kiebler MA, Bassell GJ (2006) Neuronal RNA granules: movers and makers. Neuron 51:685–690. 16982415 10.1016/j.neuron.2006.08.021

[55] Caudy AA, Myers M, Hannon GJ, Hammond SM (2002) Fragile X-related protein and VIG associate with the RNA interference machinery. Genes Dev 16:2491–2496. 12368260 10.1101/gad.1025202 PMC187452

[56] Ishizuka A, Siomi MC, Siomi H (2002) A Drosophila fragile X protein interacts with components of RNAi and ribosomal proteins. Genes Dev 16:2497–2508. 12368261 10.1101/gad.1022002 PMC187455

[57] Lebeau G, DesGroseillers L, Sossin W, Lacaille JC (2011) mRNA binding protein staufen 1-dependent regulation of pyramidal cell spine morphology via NMDA receptor-mediated synaptic plasticity. Mol Brain 4:22. 21635779 10.1186/1756-6606-4-22 PMC3118231

[58] Lebeau G, Maher-Laporte M, Topolnik L, Laurent CE, Sossin W, et al (2008) Staufen1 regulation of protein synthesis-dependent long-term potentiation and synaptic function in hippocampal pyramidal cells. Mol Cell Biol 28:2896–2907. 18316402 10.1128/MCB.01844-07 PMC2293094

[59] Vessey JP, Macchi P, Stein JM, Mikl M, Hawker KN, et al (2008) A loss of function allele for murine Staufen1 leads to impairment of dendritic Staufen1-RNP delivery and dendritic spine morphogenesis. Proc Natl Acad Sci U S A 105:16374–16379. 18922781 10.1073/pnas.0804583105 PMC2567905

[60] de Lucas S, Oliveros JC, Chagoyen M, Ortin J (2014) Functional signature for the recognition of specific target mRNAs by human Staufen1 protein. Nucleic Acids Res 42:4516–4526. 24470147 10.1093/nar/gku073 PMC3985646

[61] Choi J, Ababon MR, Matteson PG, Millonig JH (2012) Cut-like homeobox 1 and nuclear factor I/B mediate ENGRAILED2 autism spectrum disorder-associated haplotype function. Hum Mol Genet 21:1566–1580. 22180456 10.1093/hmg/ddr594 PMC3298280

[62] James SJ, Shpyleva S, Melnyk S, Pavliv O, Pogribny IP (2013) Complex epigenetic regulation of engrailed-2 (EN-2) homeobox gene in the autism cerebellum. Transl Psychiatry 3:e232. 23423141 10.1038/tp.2013.8 PMC3590998

[63] Bot AM, Debski KJ, Lukasiuk K (2013) Alterations in miRNA levels in the dentate gyrus in epileptic rats. PLoS One 8:e76051. 24146813 10.1371/journal.pone.0076051 PMC3795667

[64] Nam J, Mah W, Kim E (2011) The SALM/Lrfn family of leucine-rich repeat-containing cell adhesion molecules. Semin Cell Dev Biol 22:492–498. 21736948 10.1016/j.semcdb.2011.06.005

[65] Wang PY, Seabold GK, Wenthold RJ (2008) Synaptic adhesion-like molecules (SALMs) promote neurite outgrowth. Mol Cell Neurosci 39:83–94. 18585462 10.1016/j.mcn.2008.05.019 PMC2602877

