# Supplementary figures and images for: Human Staufen1 Associates to MiRNAs Involved in Neuronal Cell Differentiation and is Required for Correct Dendritic Formation
Source: PLoS One. 2014 Nov 25;9(11):e113704. doi: 10.1371/journal.pone.0113704 (PMC4244161; doi:10.1371/journal.pone.0113704)

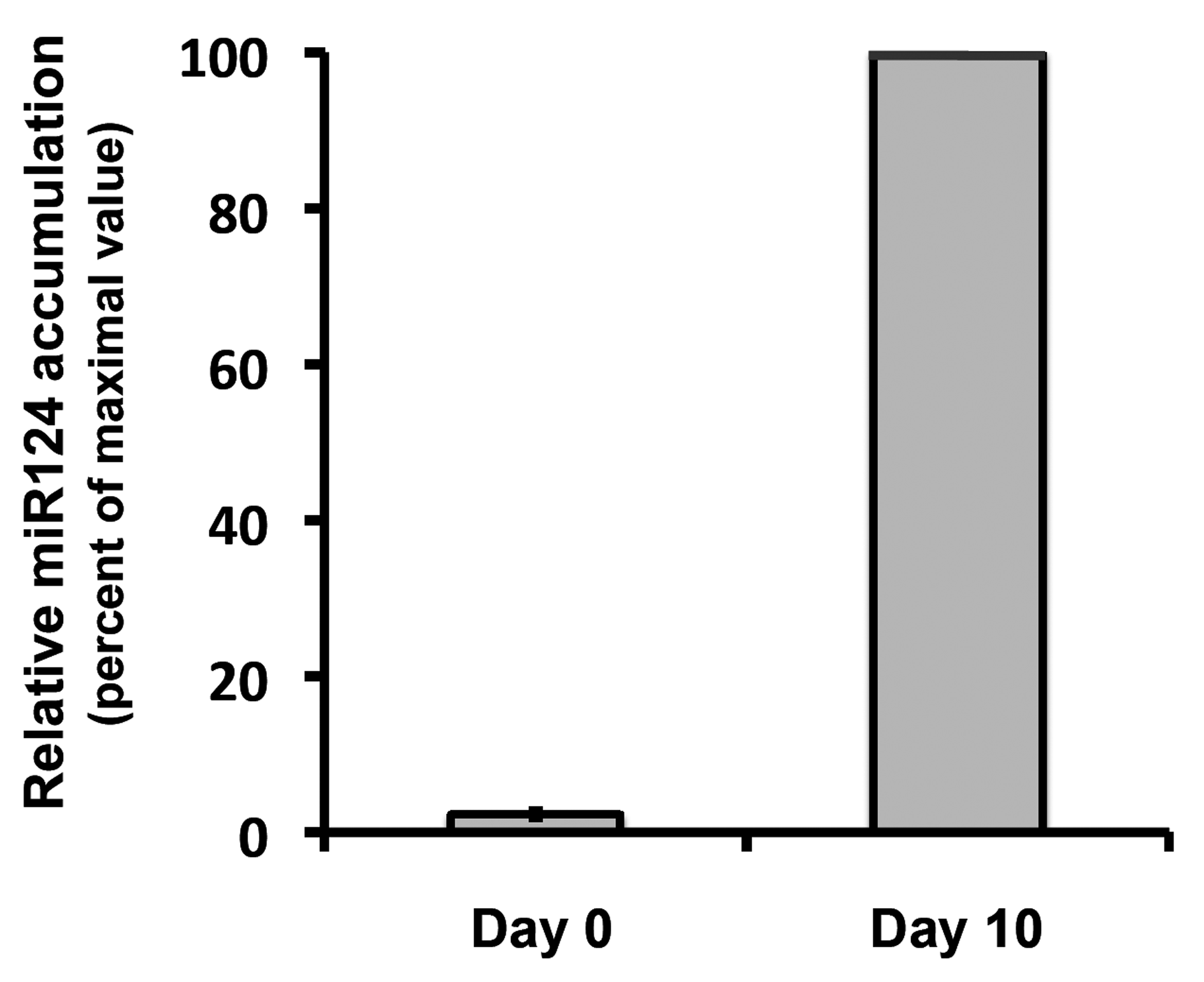

Supplement: Figure S1 — Induction of miR-124 upon neuroblast differentiation. Cultures of SH-SY5Y neuroblastoma cells were differentiated as described in Materials and Methods. Total cell extracts were isolated from cells prior to differentiation (day 0) or at a final stage of differentiation (day 10). Total cell RNA was isolated and the concentration of miR-124 was determined by TaqMan RT-qPCR. (TIF) [file pone.0113704.s001.tif]

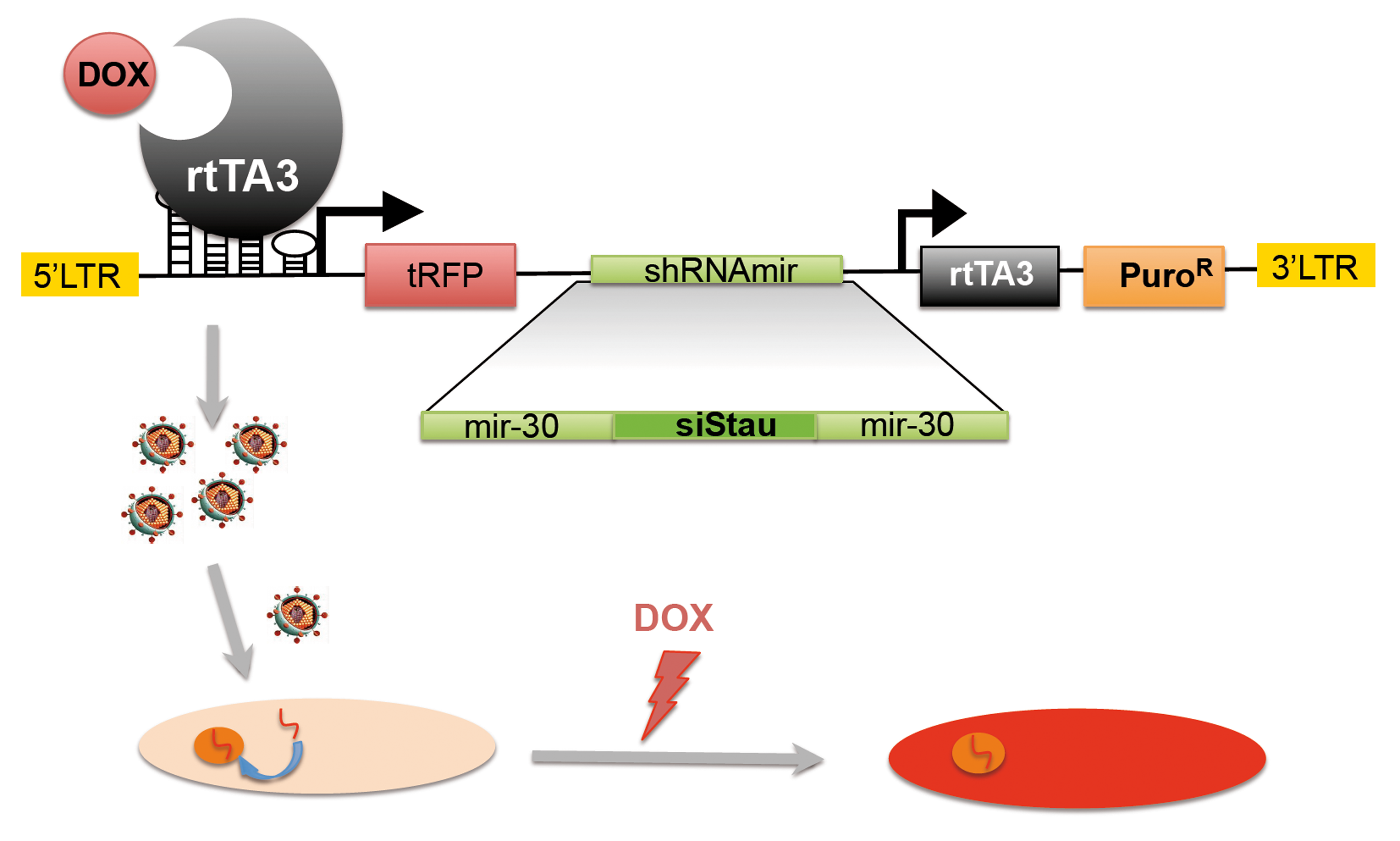

Supplement: Figure S2 — Experimental strategy for the regulated silencing of hStau1. Specific silencing sequences were inserted into the miR-30 skeleton present in pTRIPZ plasmid. The recombinant miRNA is expressed from a minimal CMV promoter under the control of Tet repressor, in a bicistronic mRNA also containing the RFP as a marker. The construct was used to generate lentiviral particles containing VSV G glycoprotein. Target cells were transduced with the recombinant lentivirus and selected with puromycin. Induction with doxicyclin leads to the expression of RFP and the hStau1 silencing RNA. (TIF) [file pone.0113704.s002.tif]

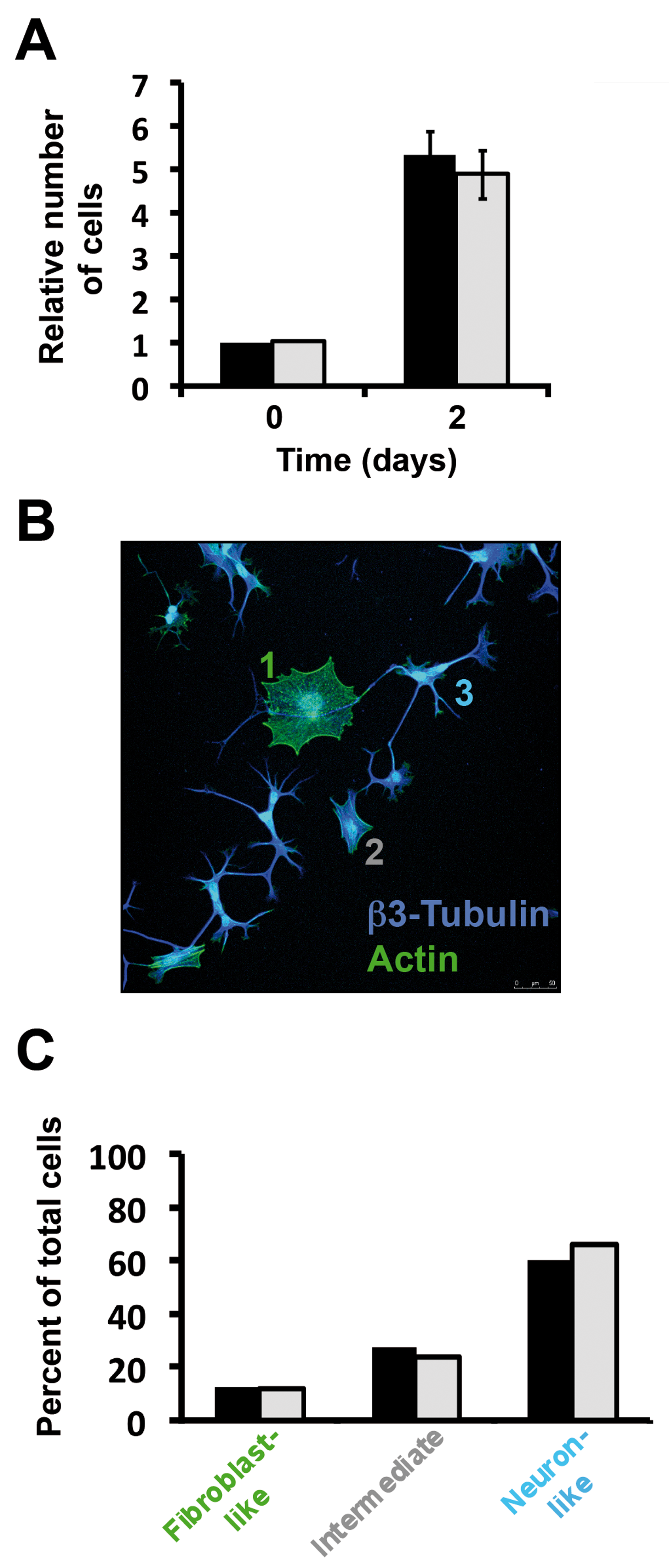

Supplement: Figure S3 — General properties of hStau1-silenced neuroblastoma cells. Cultures of SH-SY5Y neuroblastoma cells were transduced with control (black bars) or hStau1 silencing lentiviruses (grey bars). (A) The cells were cultivated and the number of cells counted. The relative number of cells at time 0 and 2 days after incubation are presented. (B) The cultures were induced for differentiation as indicated in Materials and Methods and stained to reveal ß3-tubulin and actin at day 7. A representative field of a differentiated culture is presented to show the various cell phenotypes obtained: Fibroblast-like (1; green), Intermediate (2; grey) and neuron-like (3; blue). (C) The number of cells showing the various phenotypes were counted in control (black bars) or hStau1-silenced (grey bars) cultures and is presented as percent of total cells. (TIF) [file pone.0113704.s003.tif]

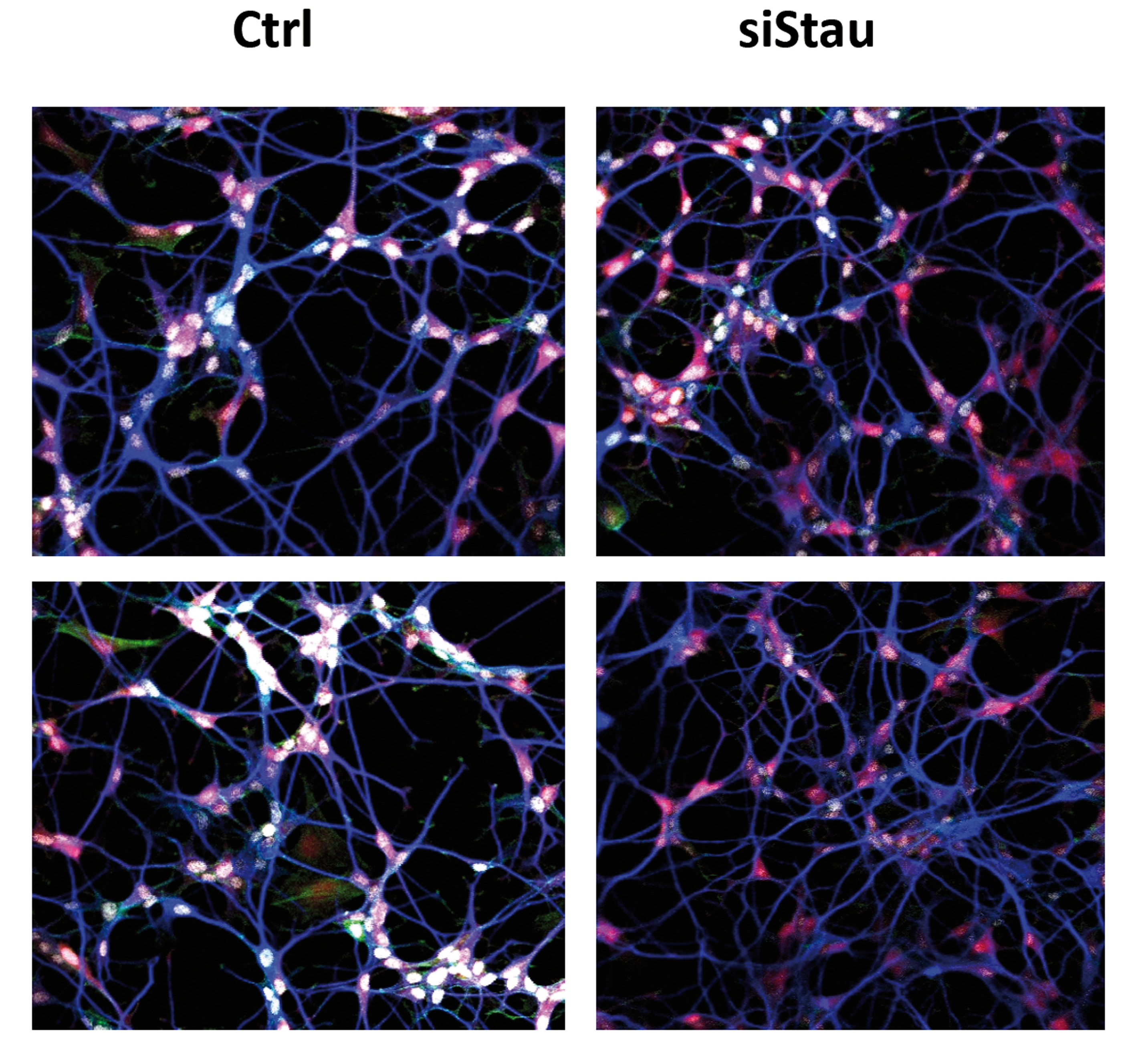

Supplement: Figure S4 — Morphology of differentiated neuroblastoma cells upon hStau1 silencing. Cultures of SH-SY5Y neuroblastoma cells previously transduced with control (Ctrl) or hStau1-specific (siStau) silencing lentiviruses were induced for differentiation as described in Materials and Methods. Starting at day 5 post-differentiation, cells were treated with doxicyclin during 5 days. From day 7 post-differentiation hStau1 expression levels were reduced. At 10 days post-differentiation the cultures were fixed and immunostained with antibodies specific for ßIII-tubulin (blue) and phalloidin (green). Red colour corresponds to the RFP signal derived from the lentiviral constructs. The images show 2 representative fields of Ctrl- or siStau-transduced cultures. (TIF) [file pone.0113704.s004.tif]
