# Supplementary material for: Human Staufen1 Associates to MiRNAs Involved in Neuronal Cell Differentiation and is Required for Correct Dendritic Formation
Source: PLoS One. 2014 Nov 25;9(11):e113704. doi: 10.1371/journal.pone.0113704 (PMC4244161; doi:10.1371/journal.pone.0113704)
Supplement: Table S1 — List of mRNAs associated to hStau1. (DOC) [file pone.0113704.s005.doc]

| **Affimetrix ID** | **Name** | **Description** | **Fold Change** |
| --- | --- | --- | --- |
| **53071_s_at** | FLJ22222 | Chromosome 17 open reading frame 101 | **4.04** |
| **228868_x_at** | CDT1 | Chromatin licensing and DNA replication factor 1 | **4.05** |
| **227667_at** | CUEDC1 | CUE domain containing 1 | **4.07** |
| **214395_x_at** | EEF1D | Eukaryotic translation elongation factor 1 delta (guanine nucleotide exchange protein) | **4.12** |
| **212496_s_at** | JMJD2B | Jumonji domain containing 2B | **4.12** |
| **218208_at** | PQLC1 | PQ loop repeat containing 1 | **4.12** |
| **47069_at** | ARHGAP8 | Rho GTPase activating protein 8 | **4.13** |
| **223394_at** | SERTAD1 | SERTA domain containing 1 | **4.13** |
| **227203_at** | FBXL17 | F-box and leucine-rich repeat protein 17 | **4.16** |
| **201827_at** | SMARCD2 | SWI/SNF related, matrix associated, actin dependent regulator of chromatin, d 2 | **4.16** |
| **212512_s_at** | CARM1 | Coactivator-asociated arginine methyltransferase 1 | **4.20** |
| **220201_at** | MNAB | Membrane associated DNA binding protein | **4.20** |
| **37005_at** | NBL1 | Neuroblastoma, suppression of tumorigenicity 1 | **4.22** |
| **238956_at** | LOC100506781 | NA | **4.23** |
| **1553960_at** | C20orf161 | Chromosome 20 open reading frame 161 | **4.27** |
| **220409_at** | CAMSAP1 | Calmodulin regulated spectrin-associated protein 1 | **4.30** |
| **222620_s_at** | DNAJC1 | DnaJ (Hsp40) homolog, subfamily C, member 1 | **4.3** |
| **218081_at** | C20orf27 | Chromosome 20 open reading frame 27 | **4.31** |
| **231017_at** | STK11 | Serine/threonine kinase 11 | **4.32** |
| **213275_x_at** | CTSB | Cathepsin B | **4.33** |
| **224715_at** | WDR34 | WD repeat domain 34 | **4.34** |
| **222206_s_at** | NCLN | Nicalin homolog (zebrafish) | **4.36** |
| **218145_at** | TRIB3 | Tribbles homolog 3 (Drosophila) | **4.40** |
| **90265_at** | CENTA1 | Centaurin, alpha 1 | **4.41** |
| **202894_at** | EPHB4 | EPH receptor B4 | **4.42** |
| **1555894_s_at** | MTSS1L | Metastasis suppressor 1-like | **4.53** |
| **229269_x_at** | SSBP4 | Single stranded DNA binding protein 4 | **4.53** |
| **219983_at** | HRASLS | HRAS-like suppressor | **4.58** |
| **225699_at** | C7orf40 | Chromosome 7 open reading frame 40 | **4.62** |
| **230972_at** | ANKRD9 | Ankyrin repeat domain 9 | **4.64** |
| **224576_at** | ERGIC1 | Endoplasmic reticulum-golgi intermediate compartment (ERGIC) 1 | **4.64** |
| **224598_at** | MGAT4B | Mannosyl (alpha-1,3-)-glycoprotein beta-1,4-N-acetylglucosaminyltransferase, isozyme B | **4.65** |
| **202329_at** | CSK | c-src tyrosine kinase | **4.66** |
| **227994_x_at** | C20orf149 | Chromosome 20 open reading frame 149 | **4.67** |
| **214149_s_at** | ATP6V0E | ATPase, H+ transporting, lysosomal 9kDa, V0 subunit e | **4.69** |
| **203103_s_at** | PRPF19 | PRP19/PSO4 pre-mRNA processing factor 19 homolog (S. cerevisiae) | **4.72** |
| **209037_s_at** | EHD1 | EH-domain containing 1 | **4.79** |
| **49452_at** | ACACB | Acetyl-Coenzyme A carboxylase beta | **4.90** |
| **233571_x_at** | C20orf149 | Chromosome 20 open reading frame 149 | **4.90** |
| **222288_at** | NA | NA | **4.92** |
| **213813_x_at** | FTL | Ferritin, light polypeptide | **4.93** |
| **217118_s_at** | C22orf9 | Chromosome 22 open reading frame 9 | **4.94** |
| **213587_s_at** | ATP6V0E2 | ATPase, H+ transporting V0 subunit e2 | **5.08** |
| **212303_x_at** | KHSRP | KH-type splicing regulatory protein (FUSE binding protein 2) | **5.08** |
| **206298_at** | ARHGAP22 | Rho GTPase activating protein 22 | **5.12** |
| **217598_at** | CINP | NA | **5.19** |
| **214021_x_at** | ITGB5 | Integrin, beta 5 | **5.22** |
| **224477_s_at** | NUDT16L1 | Nudix (nucleoside diphosphate linked moiety X)-type motif 16-like 1 | **5.23** |
| **223677_at** | ATG10 | ATG10 autophagy related 10 homolog (S. cerevisiae) | **5.29** |
| **213183_s_at** | CDKN1C | Cyclin-dependent kinase inhibitor 1C (p57, Kip2) | **5.38** |
| **50376_at** | ZNF444 | Zinc finger protein 444 | **5.39** |
| **212155_at** | RNF187 | Ring finger protein 187 | **5.46** |
| **33736_at** | STOML1 | Stomatin (EPB72)-like 1 | **5.46** |
| **206397_x_at** | GDF1 | Growth differentiation factor 1 | **6.01** |
| **205449_at** | SAC3D1 | SAC3 domain containing 1 | **6.01** |
| **241937_s_at** | WDR4 | WD repeat domain 4 | **6.01** |
| **225969_at** | ALKBH6 | AlkB, alkylation repair homolog 6 (E. coli) | **6.10** |
| **43544_at** | THRAP5 | Thyroid hormone receptor associated protein 5 | **6.22** |
| **223284_at** | NAT14 | N-acetyltransferase 14 (GCN5-related, putative) | **6.46** |
| **236626_at** | ALG1 | Asparagine-linked glycosylation 1 homolog (yeast, beta-1,4-mannosyltransferase) | **6.78** |
| **225329_at** | FAM195B | Family with sequence similarity 195, member B | **6.78** |
| **225954_s_at** | MIDN | Midnolin | **7.15** |
| **50221_at** | TFEB | Transcription factor EB | **7.36** |
| **33778_at** | TBC1D22A | TBC1 domain family, member 22A | **7.93** |
| **230699_at** | PGLS | 6-Phosphogluconolactonase | **9.87** |
| **210683_at** | NRTN | Neurturin | **13.49** |
